# Circuit-level modeling of prediction error computation of multi-dimensional features in voluntary actions

**DOI:** 10.3389/fncom.2025.1551555

**Published:** 2025-09-29

**Authors:** Yishuang Huang, Yiling Li

**Affiliations:** ^1^School of Mathematical Science, Zhejiang University, Hangzhou, China; ^2^CAEP Software Center for High Performance Numerical Simulation, Chengdu, China

**Keywords:** prediction error, prediction, actual stimuli, feature selectivity, voluntary actions, experience-dependent plasticity, multi-dimensional sensory features, neural circuits

## Abstract

**Introduction:**

Predictive processing posits that the brain minimizes discrepancies between internal predictions and sensory inputs, offering a unifying account of perception, cognition, and action. In voluntary actions, it is thought to suppress self-generated sensory outcomes. Although sensory mismatch signals have been extensively investigated and modeled, mechanistic insights into the neural computation of predictive processing in voluntary actions remain limited.

**Methods:**

We developed a computational model comprising two-compartment excitatory pyramidal cells (PCs) and three major types of inhibitory interneurons with biologically realistic connectivity. The model incorporates experience-dependent inhibitory plasticity and feature selectivity to shape excitation-inhibition (E/I) balance. We then extended it to a two-dimensional prediction-error (PE) circuit in which each PC has two segregated, top-down modulated dendrites-each bell-tuned to a distinct feature-enabling combination selectivity.

**Results:**

The model reveals that top-down predictions can selectively suppress PCs with matching feature selectivity via experience-dependent inhibitory plasticity. This suppression depends on the response selectivity of inhibitory interneurons and on balanced excitation and inhibition across multiple pathways. The framework also accommodates predictions involving two independent features.

**Discussion:**

By combining biological connectivity data with computational modeling, this study provides insights into the neural circuits and computations underlying the active suppression of sensory responses in voluntary actions. These findings contribute to understanding how the brain generates and processes predictions to guide behavior.

## 1 Introduction

Predictive processing theory provides a foundational framework for understanding cognitive and behavioral brain functions by proposing that the brain is more like an active agent than a passive information receiver (Bubic et al., 2010; Clark, 2013). This theory posits that the brain continuously generates predictions about incoming sensory information and compares these predictions with actual sensory inputs (Friston, 2005; Rao and Ballard, 1999). Central to this process is the “prediction error” (PE), which represents the mismatch between predicted and actual input (Bastos et al., 2012; Keller and Mrsic-Flogel, 2018). PE is thought to drive learning and neural plasticity, refining the brain's predictive models and improving its ability to process realworld information (Friston, 2010; Spratling, 2017). While numerous studies have identified neural signatures of prediction errors across various sensory modalities (Den Ouden et al., 2012; Auksztulewicz and Friston, 2016), our understanding of how these processes underpin voluntary actions in the brain remains incomplete.

In voluntary actions, predictive processing is critical for suppressing selfgenerated sensory outcomes (Blakemore et al., 1998; Wolpert et al., 2011). This active suppression is essential for enabling the brain to anticipate and diminish the impact of sensations resulting from its own actions, thereby distinguishing them from external stimuli (Wolpert et al., 1995). In some brain regions, it involves generating predictions based on efference copies of motor commands to anticipate the sensory feedback (Blakemore et al., 2000). Unlike PE computation in sensory systems, however, active suppression in voluntary actions generates sensory predictions that precede action execution, suggesting the involvement of distinct neural processing mechanisms (Adams et al., 2013).

Studies in somatosensory systems, for example, have illustrated that selfgenerated touch is perceived as less intense than externally generated touch of the same intensity, a phenomenon linked to reduced activity in the somatosensory cortex and cerebellum during self-generated touch (Blakemore et al., 1998; Kilteni and Ehrsson, 2020). In the auditory domain, self-generated sounds also elicit smaller neural responses compared to externally generated sounds, as demonstrated by both EEG and fMRI studies (Martikainen et al., 2005; Rummell et al., 2016). In visual systems, predictive processing has been extensively studied during eye movements, where the brain predicts and compensates for the visual displacement to maintain perceptual stability (Wurtz, 2008). This involves corollary discharge signals from the superior colliculus to the frontal eye fields and other cortical areas (Sommer and Wurtz, 2008). Recent research has extended these findings to the vestibular system and found that self-generated head movements lead to attenuated vestibular responses compared to passive movements, suggesting a predictive mechanism in vestibular processing (Cullen, 2019). Neuroimaging and electrophysiological studies have identified key brain regions involved in predictive processing during voluntary actions, including the cerebellum, parietal cortex, and prefrontal areas (Wolpert et al., 2011; Schwartze et al., 2012; Kilteni and Engeler, 2020). However, further research is still needed to fully characterize the underlying neural circuits, as well as the temporal dynamics and hierarchical organization of these predictive mechanisms in voluntary actions (Pezzulo et al., 2015; Heilbron and Chait, 2018).

In the present study, we investigated the circuit-level mechanisms underlying the computation of prediction error (PE) for both one- and two-dimensional stimulus features using a computational model with biologically realistic connectivity motifs. Our goal was to elucidate how inhibitory plasticity and interneuron selectivity jointly facilitate the emergence of PE neurons and support predictive processing during voluntary behavior. The model incorporates feature-selective pyramidal cells (PCs) along with three major classes of inhibitory interneurons-parvalbumin-positive (PV), somatostatin-positive (SOM), and vasoactive intestinal peptide-positive (VIP) neurons and employs experience-dependent inhibitory plasticity to dynamically establish excitation-inhibition (E/I) balance across neuronal compartments. Crucially, we extended feature selectivity to inhibitory populations and revealed that selective inhibition is essential for prediction-dirven suppression. Our results demonstrate that the model gives rise to PE neurons exhibiting hallmark computational properties of predictive coding: (1) mismatch responses scale with the degree of prediction violation; (2) top-down and bottom-up signals are integrated in a subtractive manner; and (3) feature-specific mismatch responses are selectively amplified with experience. Furthermore, the compartmentalized dendritic architecture naturally supports the extension to multidimensional features, enabling scalable and functionally specific PE computation. Our results suggest that the relationship between voluntary behavior and prediction error neurons for multidimensional features is intricately linked to the brain's ability to adapt and respond to complex environments.

More broadly, voluntary behavior involves the self-initiated execution of actions based on internal goals and motivations, often requiring anticipation and prediction of future events. Prediction error neurons for multidimensional features play a crucial role in this process by constantly comparing expected outcomes with actual sensory inputs. These neurons detect discrepancies between predicted and observed stimuli, signaling the need for adjustments in the brain's internal model of the environment. By encoding not only the magnitude but also various dimensions of sensory features such as direction, color, and location, these neurons provide nuanced feedback to guide behavior. In summary, multidimensional prediction error neurons offer a functional framework linking predictive processing to the generation of voluntary behavior in complex environments.

## 2 Results

We employed a four-population firing rate model to simulate microcircuit activity and explored the development of prediction error (PE) neurons in the context of voluntary behavior. The network model comprises excitatory pyramidal cells (PCs) alongside inhibitory cells categorized into three major types based on their protein expression: parvalbumin (PV), somatostatin (SOM), and vasoactive intestinal peptide (VIP) neurons (Rudy et al., 2011). To enhance the biological realism and account for SOM interneuron heterogeneity, we included two subclasses of SOM neurons: Martinotti (M) cells and non-Martinotti (nM) cells (Ma et al., 2006). These subclasses exhibit distinct synaptic targeting patterns, with Martinotti cells targeting pyramidal neuron dendrites (Rudy et al., 2011; Jiang et al., 2015; Kawaguchi and Kubota, 1997) and non-Martinotti cells targeting the soma (McGarry et al., 2010). Inhibitory neurons were implemented as point neurons based on the model proposed by Wilson and Cowan (1972). For PC neurons, we employed a reduced multi-compartmental neuron model consisting of one somatic compartment and multiple dendritic compartments. To model one-dimensional feature PE neurons, we initially considered a single dendritic branch and later extended the model to accommodate two-dimensional features by including two dendritic branches. In this configuration, each pyramidal neuron receives converging inputs from both dendritic branches, each carrying distinct stimulus features, resulting in selectivity for a preferred stimulus along each feature dimension. This dendritic morphology is motivated by prior studies showing that distinct dendritic branches can independently integrate localized inputs (Poirazi et al., 2003).

Simple cells in the primary visual cortex (V1) exhibit selectivity to various stimulus properties, such as color, orientation, motion direction, and stimulus location. In our model, we replicated the stimulus-tuning observed in pyramidal cells (PCs) in layer 2/3 of mouse V1 by providing each of the 280 excitatory neurons and 40 SOM neurons with external input tuned using one- or two-dimensional Gaussian functions, consistent with experimental findings (Ma et al., 2010; Niell and Stryker, 2008). The preferred stimuli of PCs and SOM neurons were distributed evenly across the stimulus space. We initially focused on one-dimensional stimuli, restricting the stimulus feature space to the discrete values 0, 1, 2, and 3, which could also represent features like direction and color through appropriate mapping (Li, 2023). Within this context, there are four types of PC neurons with different preferred stimuli. In our framework, predictions and actual sensory inputs are encoded as stimulus features, with prediction errors quantified as the differences between the predicted and observed features. Notably, the PCs in this network generally exhibit specific stimulus selectivity (here, one of four types), either in one or two dimensions, and each selective group is considered homogeneous. Accordingly, these groups are analyzed separately.

In the model, all neurons receive excitatory background input to maintain reasonable baseline activity levels. This ensures that even in the absence of visual input or motor-related internal predictions, the neurons remain active and ready to process incoming signals. The network is also stimulated with time-varying external inputs representing actual and predicted visual stimuli. Sensory stimuli are presented with intervening pauses (background phases), analogous to gaps between stimuli in sensory sequences (e.g., blank screens in vision). We hypothesize that sensory consequences of voluntary movements are fully predicted by internal motor commands (“match phase,” *P* = *S*). Conversely, unexpected external changes or mismatched sensory feedback generate unpredicted signals (“mismatch phase,” *P*≠*S* ). The circuit we studied was motivated by the widely accepted view that PCs, PV, and SOM interneurons in V1 exhibit visually driven activity (Ko et al., 2011; Yang et al., 2013; Harris and Shepherd, 2015; Xue et al., 2014; Lee et al., 2016; Larkum, 2013). In contrast, long-range (e.g., motor) predictions target VIP neurons (Fu et al., 2014; Ibrahim et al., 2016; Attinger et al., 2017) and the apical/dendritic compartments of PCs (Attinger et al., 2017; Larkum, 2013) in superficial V1 layers. Within this framework, VIP neurons act as key disinhibitory elements, suppressing SOM and PV interneurons in response to predictive feedback (Pi et al., 2013; Zhang et al., 2014), thereby modulating PC activity context-dependently.

Although parvalbumin-expressing (PV) interneurons also inhibit PCs in the network, they were modeled as receiving untuned sensory input. This assumption is supported by experimental evidence that PV interneurons form dense, nonspecific connections and provide broad, unselective inhibition to nearby excitatory neurons (Packer and Yuste, 2011). In contrast, SOM interneurons exhibit significantly higher stimulus selectivity, particularly in direction tuning, as demonstrated by Kerlin et al. (2010), making them well-suited for implementing feature-specific inhibition. Furthermore, since our model is designed to realize prediction-driven selective suppression, the role of SOM neurons becomes especially critical during phases when only top-down predictive inputs are present. In such cases, dendritic compartments of PCs become the primary source of somatic excitation. To counteract this excitation in a stimulus-specific manner, it is essential that SOM neurons deliver selective inhibition. Therefore, incorporating stimulus-tuned SOM neurons that specifically target PC dendrites is likely essential for enabling prediction-dependent, feature-specific suppression.

**Figure 1:**
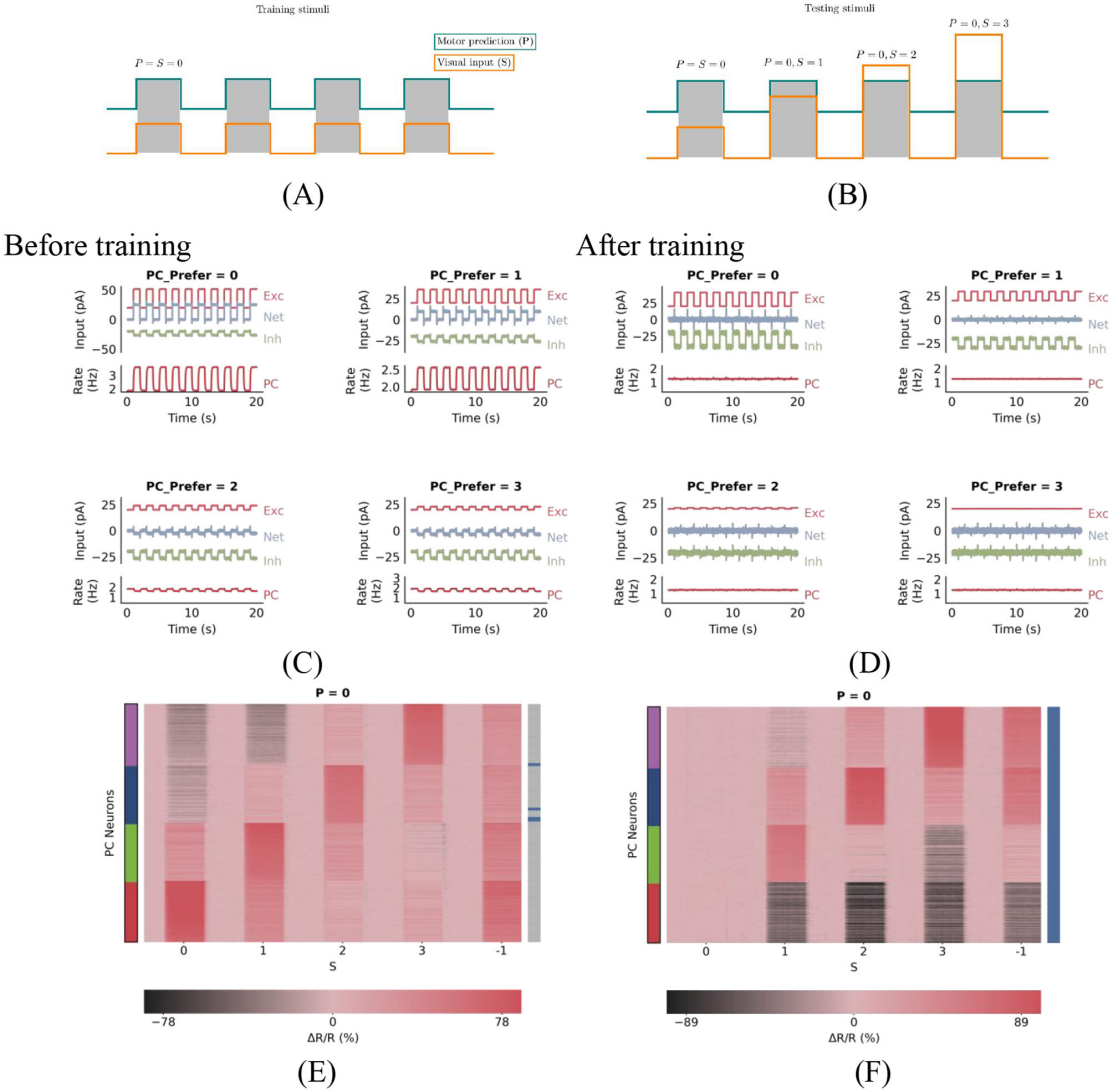
PC neuron activity in response to stimuli before and after training. **(A)** During plasticity, the network undergoes a series of match phases, representing coupled sensorimotor experiences and referred to as training stimuli. **(B)** Test stimuli consist of mismatch phases, during which visual inputs deviate from motor predictions. Each stimulus is presented for 1 s and alternates with baseline phases, during which both visual inputs and motor predictions are absent. **(C**, **D)** Excitation-inhibition (E/I) balance before **(C)** and after **(D)** training. **(C)** Before plasticity, the somatic excitation and inhibition in PCs are not balanced, leading to deviations in the population firing rate from baseline. **(D)** After plasticity, somatic excitation and inhibition are balanced, keeping the PC population rate stable at baseline. **(E**, **F)** Prediction-error neurons emerge through E/I balance. These panels show PC neuron responses to test stimuli before **(E)** and after **(F)** training. The panel title denotes the prediction *P*, while the horizontal axis represents the actual stimulus *S*. The vertical axis lists *N*_PC_ PC neurons, with color-coded strips indicating their preferred stimuli: red, green, blue, and purple denote preferences for stimuli 0, 1, 2, and 3, respectively. Blue horizontal lines in the right bar indicate PC neurons exhibiting prediction-error (PE) neuron properties: during 0-type match phases, PE neurons maintain baseline activity, while during 0−*S* mismatch phases, neurons preferring stimulus *S* show significant excitation. As shown in the right bar of panel (F), all PCs are classified as prediction-error neurons after training.

In this network, PE neurons are defined as excitatory cells that remain at baseline in the match phase and maintain a certain amount of activity in the corresponding mismatch phase. Specifically, we classified PCs as PE neurons when the change in firing rate, normalized by baseline firing rate (Δ*R*/*R* = *r*−*r*_BL_/*r*_BL_), exceeds a threshold 20% during the corresponding mismatch phase and is less than 10% during the match phase. Allowing for minor deviations in the match and mismatch phases aligns more closely with experimental methods. The specific threshold values are not crucial to the results. For instance, after training with matched stimuli as shown in Figure 1A, PE neurons preferring stimulus ‘0' remain at baseline activity during the 0-type match phase (‘0 − 0', where the former represents predicted stimuli and the latter represents actual stimuli) of the test stimulus in Figure 1B, while those preferring stimulus ‘1' also maintain baseline activity in this phase (all selective neurons perceive this as a match), yet exhibit the highest activity during the ‘0 − 1' mismatch phase among all neurons.

### 2.1 One-dimensional prediction error neurons emerge by balancing excitation and inhibition

Before training, somatic excitation in PCs was not balanced by inhibition (Figure 1C), leading to elevated activity even during matched input conditions. After training, somatic E/I balance was achieved (Figure 1D). This balance is crucial for accurate prediction error computation and emerges dynamically through synaptic plasticity (Hertäg and Sprekeler, 2020).

Initially, the neural circuit had randomly initialized synaptic connections, resulting in an imbalance between excitation and inhibition among PCs. As a result, all PCs showed changes in firing rates in response to matched stimuli, indicating the absence of PE neurons (Figure 1E). During simulated sensorimotor experience, inhibitory plasticity gradually adjusted inhibitory synapses to minimize deviations of PC firing rates from baseline levels (Figure 1D). After training, all neurons showed minimal response to matched stimuli (Figure 1F), consistent with PE neuron behavior. Thus, inhibitory synaptic plasticity plays a critical role in generating PE neurons by balancing excitation and inhibition in PCs during plasticity.

**Figure 2:**
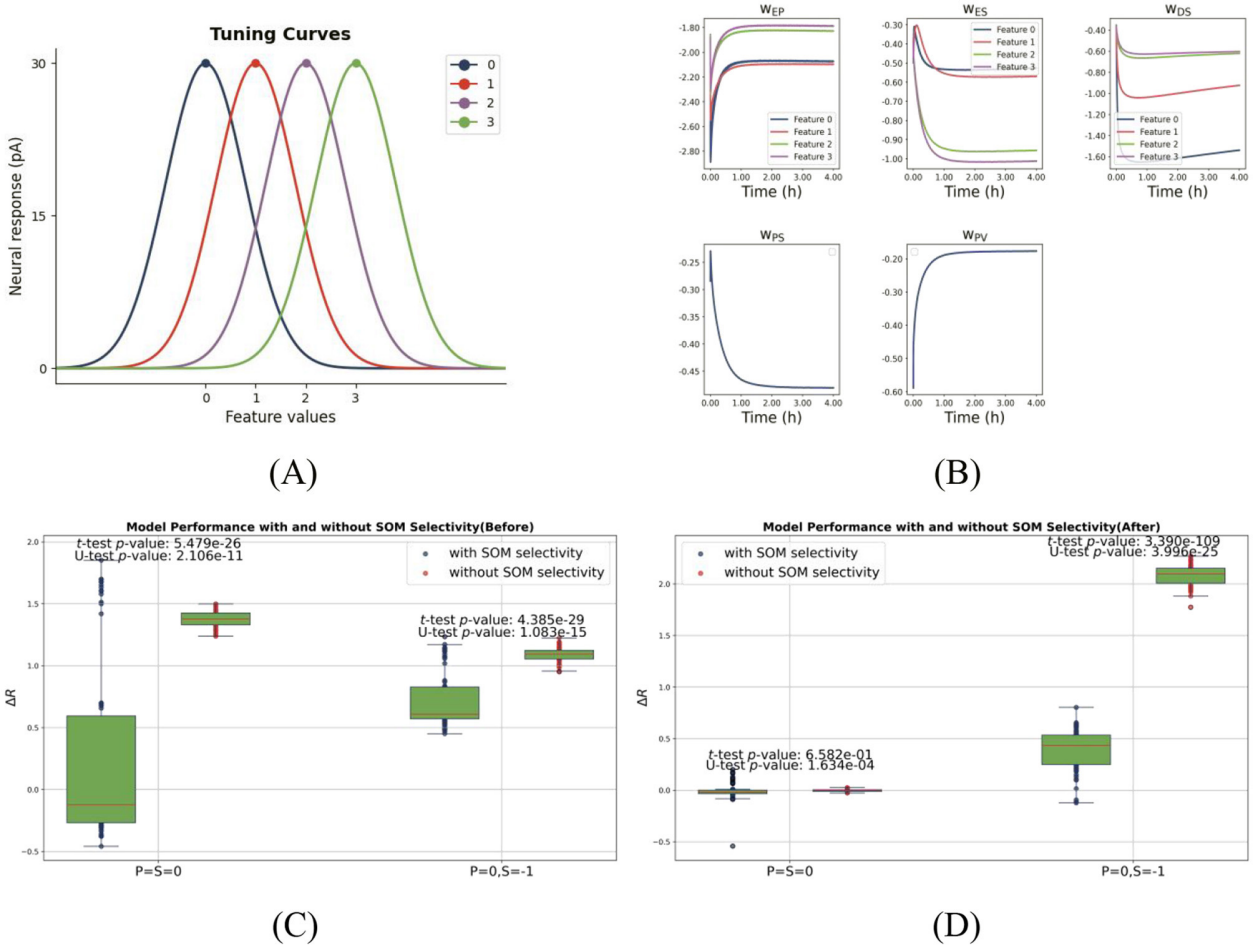
Feature selectivity and network performance with and without SOM selectivity. **(A)** Gaussian tuning curves for one-dimensional feature selectivity. The network comprises four types of pyramidal cells (PCs), each with a distinct stimulus preference, represented by differently colored Gaussian profiles. PCs exhibit narrow tuning (σ_PC_ = 0.8), while SOM neurons (when selective) show broader tuning (σ_SOM_ = 1), consistent with experimental observations. **(B)** Inhibitory plasticity drives synaptic weight differentiation during training, allowing PC firing rates to converge to the target and enabling prediction-driven selective suppression. Network performance before **(C)** and after **(D)** training, comparing models with SOM selectivity (blue) and without (red). Each panel shows two pairs of boxplots for PCs preferring “0” across two test conditions: (1) matched input (*P* = *S* = 0); (2) predictive input only (*P* = 0, *S* = −1). Dots represent individual PCs. Statistical significance (*p* < 0.05, two-sample *t*-test and Mann-Whitney U-test) indicates that SOM selectivity significantly enhances predictive suppression. This figure is designed to evaluate how SOM neuron selectivity influences two critical mechanisms in the one-dimensional model: (1) excitation-inhibition (E/I) balance under matched stimuli and (2) selective suppression in the presence of top-down prediction alone.

Our computational model demonstrates that PE neurons encoding either one- or two-dimensional stimulus features (Figure 2A for the one-dimensional case) can emerge through such E/I balancing mechanisms in cortical circuits. Additionally, our study provides further insights into the circuit-level implementation of prediction-error computation, as described in the following sections.

### 2.2 Inhibitory plasticity and tuned inhibitory neurons support selective suppression by top-down prediction

To understand how inhibitory plasticity and feature-selective inhibition enable feature-specific suppression, we systematically examined synaptic dynamics and PC responses in models with and without SOM neuron selectivity.

We first assessed the effect of inhibitory plasticity with predictive input alone. Before training (homogeneous weights), top-down input failed to induce feature-specific PC suppression (Figure 1E, rightmost column, γ(0) = 0). After training, PCs exhibited differentiated suppression, with neurons tuned to the predicted stimulus (e.g., “0”) being strongly inhibited. This demonstrates successful prediction-driven selective suppression. This effect was quantified using the selectivity coefficient (Equation 12, Materials and Methods), which revealed enhanced feature-specificity after training [γ(0) = 0.65]. As Figure 2B shows, inhibitory synaptic weights onto PCs diverged during training, forming a feature-specific inhibitory landscape. This contrasts with classical attractor models implementing global inhibition (Amit and Brunel, 1997), highlighting our model's feature-dependent architecture.

We next compared networks with and without SOM selectivity as shown in Figures 2C, D. Without SOM selectivity, top-down input induced weaker, less targeted PC suppression (Supplementary Figure 1), indicating impaired inhibitory differentiation. This supports the notion that feature-specific suppression relies on tuned inhibitory populations (Li, 2023). Incorporating SOM neurons with broader tuning (σ_SOM_>σ_PC_), consistent with experimental data (Sohya et al., 2007), enhanced network performance. PCs exhibited stronger and moreselective suppression under predictive input *P* = 0, *S* = −1 (Figures 1F, 2D). Statistical comparisons confirmed significant differences (*p* < <0.05, *t* test/MWU test), particularly under predictive input (Figure 2D). Thus, SOM selectivity critically shapes feature-specific inhibition.

Finally, a study by Ma et al. (2010) demonstrated that the orientation selectivity of SOM neurons is comparable to that of pyramidal cells. To assess the impact of SOM neurons' selectivity level on our model, we conducted an additional simulation in which SOM neurons were assigned the same tuning width as pyramidal cells (σ_PC_ = σ_SOM_ = 0.8).

The results (see Supplementary Figure 2) indicate that the model's performance remained qualitatively unchanged, suggesting that the critical factor is the presence of feature selectivity itself, rather than the exact sharpness of the tuning.

In summary, our results demonstrate that both inhibitory plasticity and the feature selectivity of SOM neurons are essential for prediction-driven suppression. (1) Experience-dependent inhibitory plasticity establishes not only a stable excitationinhibition (E/I) balance, but also enables top-down predictions to selectively suppress pyramidal cell (PC) activity. (2) Feature tuning in SOM interneurons is critical for eliciting feature-specific suppression; in its absence, inhibitory modulation becomes less effective and less selective. These results underscore that both inhibitory plasticity and interneuron selectivity are indispensable for implementing prediction-based suppression and ensuring efficient sensory processing.

### 2.3 One-dimensional feature enables key properties of PE neurons

**Figure 3:**
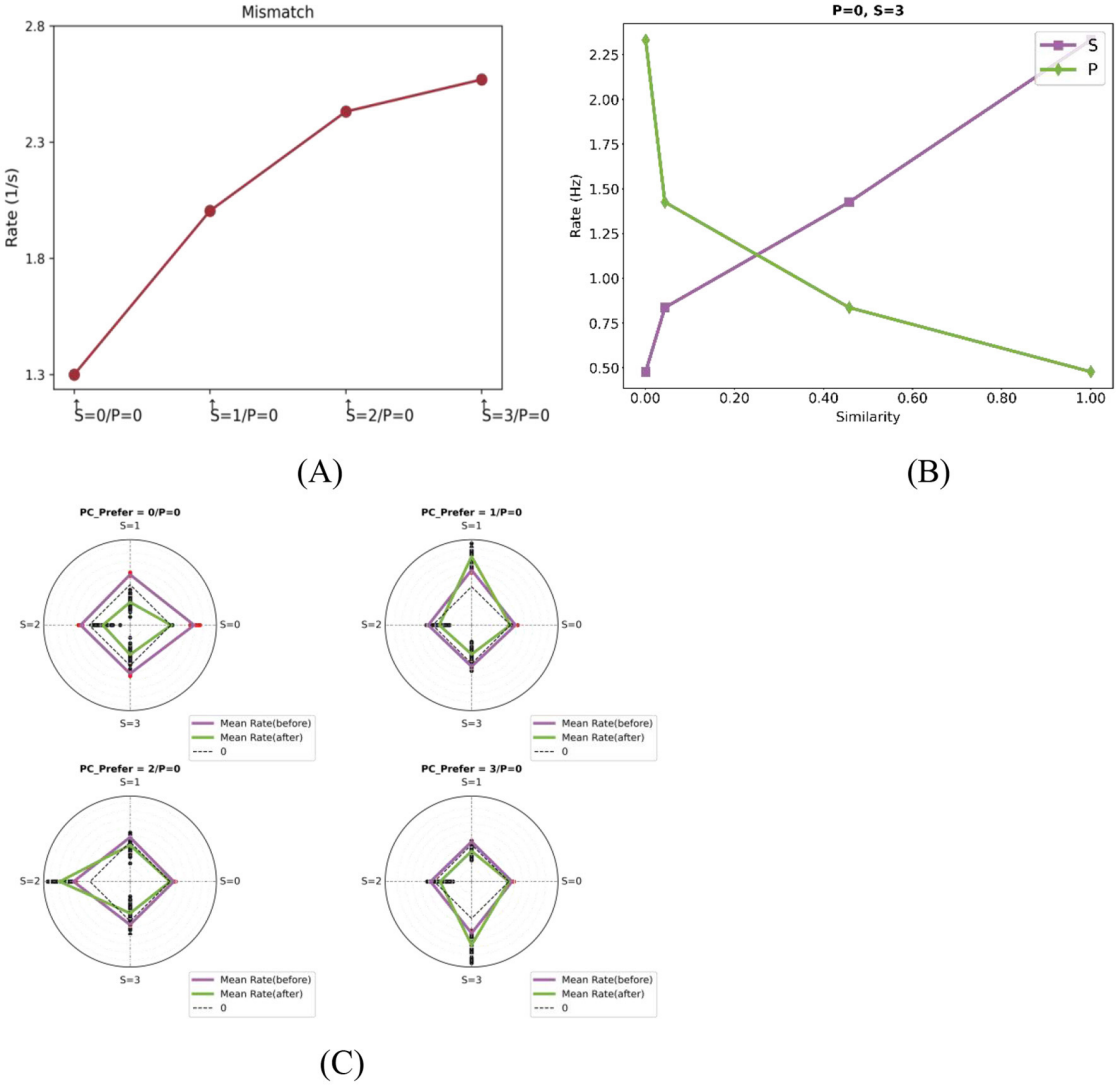
Computational properties of PE neurons with one-dimensional feature tuning. **(A)** Mismatch response magnitude scales with prediction-sensory deviation. With prediction fixed at “0,” responses show monotonic increase as the actual stimulus deviates further from this prediction. Each data point denotes the population-average response of feature-selective neurons for a corresponding actual stimulus, as indicated on the horizontal axis. For example, the second point represents the population-average response of neurons preferring stimulus “1” during the mismatch phase when *P* = 0 and *S* = 1. **(B)** Neural responses exhibit dual dependencies: they are positively correlated with the similarity to the actual stimulus (purple) and negatively correlated with the similarity to the predicted stimulus (blue). The plots show population-average responses to mismatched stimuli (as indicated in the titles) for four neurons with different selectivities. The x-axis represents the similarity between each neuron's preferred stimulus and either the actual or predicted stimulus. **(C)** Feature-specific mismatch amplification after training. Radar plots show the population-average responses of four PC subtypes to different actual stimuli under a fixed prediction condition *P* = 0 (as indicated in subplot titles, which also specify each subtype's preferred stimulus). Radial directions represent different actual stimuli Two solid lines are shown: the purple line indicates the average response before training, and the green line indicates the response after training. Red scatter points represent individual neuron responses under each condition. Notably, selective responses to preferred mismatches are enhanced after training for example, PCs preferring “1” show amplified responses in the 0–1 mismatch phase.

Mismatch negativity (MMN), a well-established EEG signature of prediction error, is typically elicited using auditory or visual oddball paradigms and shows amplitude scaling with stimulus deviance (Näätänen et al., 2007; Tiitinen et al., 1993). Our model replicates this fundamental property: PC responses increase monotonically with the feature distance between actual and predicted stimuli. To quantify this effect, we grouped deviant stimuli based on their feature distance from the predicted input and analyzed the corresponding responses of feature-selective PC neurons. As shown in Figure 3A, the response strength increases with the degree of mismatch between predicted and actual stimuli, indicating that PE neurons encode both the presence and magnitude of sensory deviations.

To dissect this computation, we analyzed how actual and predicted inputs interact to modulate neural activity. Prior theoretical studies have proposed two primary models for integrating these inputs. In divisive models, one input (typically top-down prediction) scales the effect of the other (bottom-up sensory input), effectively modulating neural gain (Spratling, 2008, 2010). In contrast, subtractive models posit that top down predictions actively cancel or suppress bottom-up inputs, with neural responses reflecting the residual mismatch (Rao and Ballard, 1999; Ayaz and Chance, 2009). As shown in Figure 3B, PE neuron responses in our model were positively correlated with the similarity between a neuron's preferred stimulus and the actual sensory input, but negatively correlated with similarity to the predicted input. This pattern suggests that PE neuron activity reflects opposing influences from sensory evidence and predictive signals—being enhanced by actual input and suppressed by prediction—thus supporting a subtractive computation scheme within the modeled circuit. Such a mechanism naturally gives rise to a graded response profile: the greater the deviation between prediction and sensory input, the larger the mismatch signal encoded by PE neurons.

In addition, some neurons exhibited feature-specific response enhancement to specific types of mismatches. As illustrated in Figure 3C, PC neurons tuned to stimuli 1, 2, and 3 showed peak responses during mismatch phases involving their preferred features (e.g., 0-1, 0-2, 0-3), both before and after training. These results indicate that PE neurons preferentially encode mismatches aligned with their tuning profiles, consistent with experimental observations in both rodent and human studies (Fiser and Mahringer, 2016; Stefanics et al., 2019). Notably, these feature-specific mismatch responses became more pronounced after training, reflecting a sharpening of mismatch tuning. This suggests that synaptic plasticity mechanisms in the model adaptively sharpen the tuning of PE neurons to mismatched inputs.

In summary, one-dimensional PE neurons exhibit three hallmark properties: (1) mismatch responses scale proportionally with the degree of deviation from prediction; (2) bottom-up sensory input and top-down predictions exert opposing influences on activity, consistent with subtractive computation; and (3) feature-specific mismatch responses are selectively enhanced through experience-driven plasticity. Together, these properties enable PE neurons not only to detect the presence of prediction errors, but also to encode their magnitude and feature content in a dynamically adaptive mannerhallmarks of predictive coding frameworks.

### 2.4 Training preserves the original stimulus tuning of PC neurons

**Figure 4:**
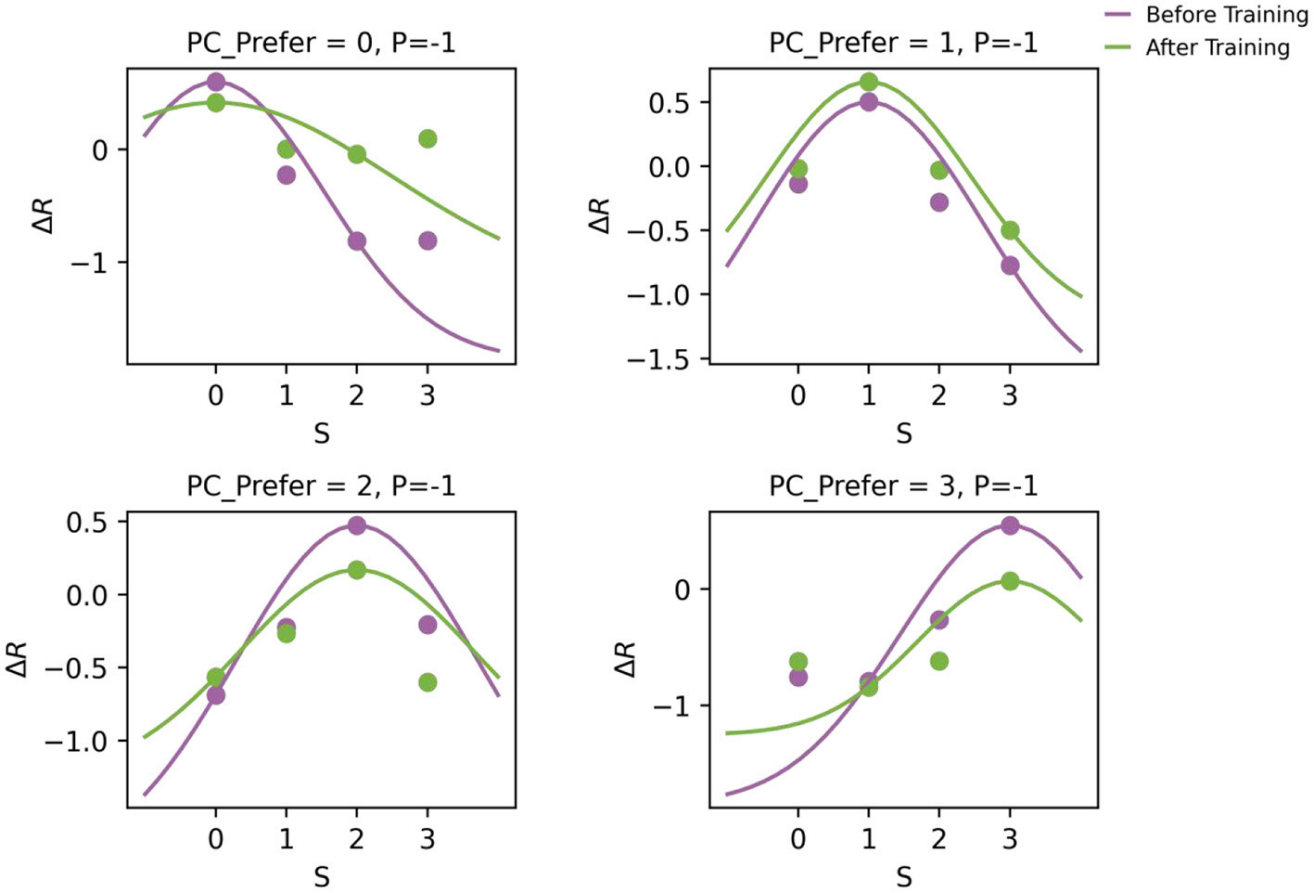
Tuning curves of PC neurons before and after training in the one-dimensional feature prediction error model. Each subplot displays the tuning curves of a PC subgroup with a specific preferred stimulus (as indicated in the title). Scatter points represent the population-average steady-state responses to different input stimuli (x-axis), and solid lines show their corresponding fitted Gaussian tuning curves. Notably, the preferred stimulus (tuning peak) remains stable after training, indicating that the emergence of PE neurons does not compromise the intrinsic feature selectivity of PC neurons.

To ensure biological plausibility, it is critical that pyramidal (PC) neurons retain their original stimulus tuning after training - even as they acquire the capacity to signal prediction errors. As shown in Figure 4, the tuning curves of PC neurons remained stable before and after training. Although the overall firing rates slightly decreased, the peak positions of the tuning curves did not shift, indicating that the emergence of prediction error (PE) circuitry did not alter neurons' inherent stimulus preferences (Li, 2023). This finding aligns with experimental observations that locomotion can modulate but not change the visual selectivity of V1 neurons (Niell and Stryker, 2010).

Furthermore, the model's enhanced response to unexpected stimuli (Section 2.3) arises exclusively from amplified prediction-sensory mismatch encoding, not from increased tuning to the stimulus itself (e.g., receptive field sharpening). This dissociation supports the theoretical framework in which predictions and errors utilize distinct neural codes. Predictions are conveyed through feature-specific activity patterns, consistent with experimental evidence that sensory templates can be pre-activated prior to stimulus onset (Kok et al., 2017). In contrast, prediction errors are encoded as deviations from these expectations. This architecture enables flexible adaptation to unexpected events while maintaining stable sensory representations — a core requirement for predictive processing.

### 2.5 Scalable prediction error computation for multi-dimensional features

**Figure 5:**
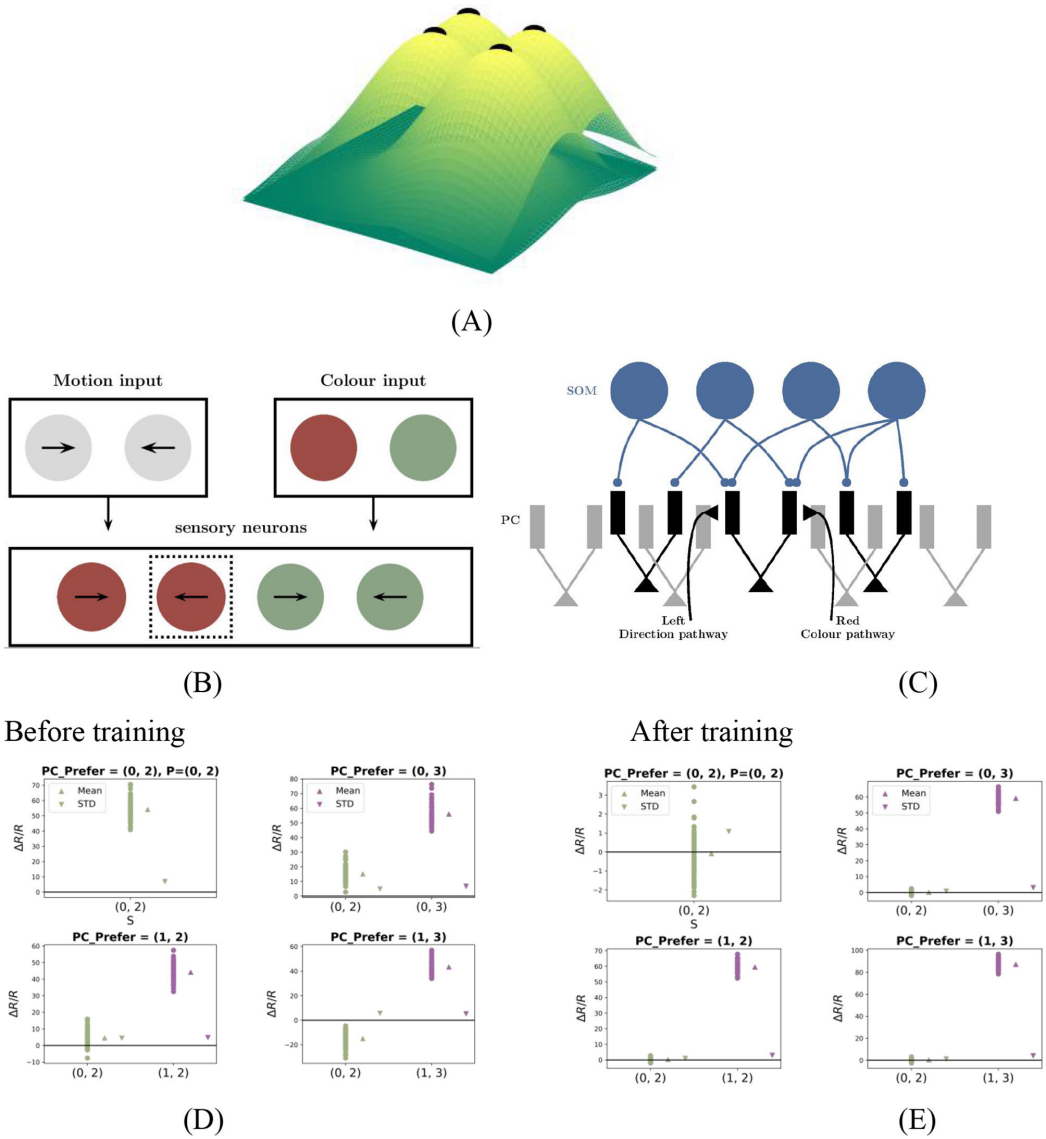
Two-dimensional PE neuron model and performance. **(A)** Two-dimensional Gaussian tuning surfaces for PCs with preferred stimuli: e.g., (0, 2), (0, 3), (1, 2), (1, 3). **(B**, **C)** Pathway-specific model structure. Motion- and color-related pathways converge on segregated dendritic compartments, modulated by dendrite-targeting interneurons. **(D**, **E)** PC responses before and after training with (0, 2)−(0, 2)stimuli. Each panel contains four subplots, corresponding to PC subgroups with different preferred stimuli. In all subplots, the prediction input is fixed at P = (0, 2), while the actual stimulus varies along the x-axis. Each dot represents the steady-state response of an individual PC neuron. “Mean” denotes the population average, and “STD” indicates variability across neurons. After training, PCs exhibit key properties of two-dimensional prediction-error (PE) neurons: (1) Under matched condition [e.g., *P* = *S* = (0, 2)]: responses remain near baseline. (2) Under mismatched conditions [e.g., *P* = (0, 2), *S* = (1, 2)]: neurons tuned to the actual stimulus [e.g., (1, 2)] show strong activation. This figure tests the model's ability to generalize to a two-dimensional feature space and demonstrates the emergence of PE neurons that exhibit hallmark mismatch responses along both dimensions.

In natural environments, sensory stimuli often vary along multiple dimensions. For instance, in the visuomotor system, head movement (a top-down motor signal) covaries with changes in the visual scene (a bottom-up input), and prediction errors emerge when this correspondence is disrupted. we extended our model to two-dimensional prediction error (PE) neurons using a two-dimensional Gaussian surface, as shown in Figure 5A.

Each neuron comprises multiple functionally segregated dendritic compartments that independently receive inputs from distinct feature pathways and interact solely through the soma, with no lateral dendritic communication (Yang et al., 2016). As a representative case, we constructed a two-dimensional model, illustrated in Figures 5B, C, where two dendritic compartments, respectively encode features such as direction and color, each modulated by top-down predictions. These inputs are modeled with bellshaped tuning curves, and their somatic integration enables PC neurons to selectively respond to specific feature combinations across both dimensions.

In our two-dimensional simulation, the first feature ℱ_1_ was limited to values {0, 1}, and the second feature ℱ_2_ to values {2, 3}, yielding four PC neuron subpopulations tuned to preferred pairs: (0, 2), (0, 3), (1, 2), and (1, 3). The response logic of these two-dimensional PE neurons closely parallels that of one-dimensional counterparts. For instance, after training with (0, 2)−(0, 2) stimuli, PE neurons tuned to (0, 2) remained at baseline activity during the (0, 2)−(0, 2) match phase, whereas those preferring (0, 3) also maintained baseline activity during this phase but exhibited the strongest mismatch responses among all neurons during the (0, 2)−(0, 3) mismatch phase.

As shown in Figure 5D (before training) and Figure 5E (after training), our model extends naturally from one- to two-dimensional PE circuits. This extension preserves the key computational properties identified in the one-dimensional case, including excitation-inhibition balance, mismatch responses that scale with the degree of prediction violation, and subtractive integration of top-down and bottom-up signals (see Supplementary Figures for detailed results). Notably, this generalization is structurally straightforward, requiring only the addition of dendritic branches to accommodate each new feature dimension. Since features are processed independently (no cross-dendrite communication), the core architecture scales without functional redesign. As a result, our framework provides a scalable and biologically plausible approach for constructing multidimensional PE neurons capable of encoding complex, feature–rich sensory environments.

### 2.6 Attention as precision-weighted prediction error amplification

In predictive coding theory, attention is conceptualized as a precision-weighting mechanism that amplifies the influence of reliable prediction errors (Friston, 2005). By assigning greater weight to more reliable errors, the brain can adjust its internal models more effectively. Attention plays a central role in this process by prioritizing sensory discrepancies that are most relevant to the task or context (Smout et al., 2019; Hohwy, 2012).

**Figure 6:**
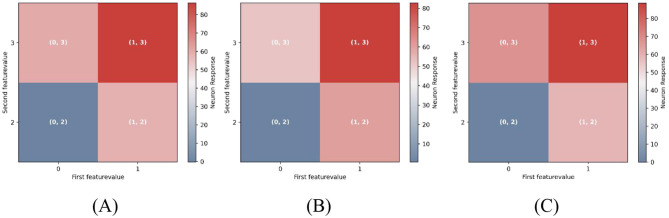
Feature-selective attention modulates multidimensional mismatch responses. Each grid cell represents the response amplitude of feature-selective pyramidal neurons during the mismatch phase, with the prediction feature fixed at (0, 2). For example, the grid cell labeled (1, 2) indicates the response of neurons preferring (1, 2) when *P* = (0, 2), *S* = (1, 2). Color intensity encodes response magnitude, with warmer colors (e.g., red) indicating stronger activation. **(A)** Neural responses under unbiased attention (equal gain across both feature dimensions). **(B)** Attention biased toward Feature 1 (first dimension) by increasing its input gain by 20%. **(C)** Attention biased toward Feature 2 (second dimension), with increased gain in the second dimension. Simultaneous mismatches across both feature dimensions elicit greater neural responses than single-feature mismatches. Moreover, attentional bias further amplifies mismatch responses for the attended feature, underscoring the role of precision-weighting in predictive coding.

To simulate attentional effects, we applied feature-specific gain modulation to the input pathways of pyramidal neurons (PCs). In the unbiased condition (Figure 6A), both feature input were assigned equal gain values (1.0). In the attention-biased conditions (Figures 6B, C), the gain for one feature pathway was increased by 20%, mimicking selective attention directed toward either the first or second dimension. Implementation details are provided in “Stimulus Selectivity” of Materials and methods.

As illustrated in Figure 6, simultaneous mismatches in both features evoke the strongest responses [e.g., Figure 6A, grid cell (1, 3)], consistent with the scaling observed in the one-dimensional model. Furthermore, attention selectively enhanced mismatch responses along the attended feature axis. Specifically, when prediction violations occurred along the attended dimension, PE neurons exhibited significantly stronger responses compared to violations along the unattended dimension. For example, in Figure 6B, mismatches along Feature 1 (rows) elicited stronger responses than those along Feature 2 (columns). This demonstrates how cortical gain modulation implements precision-weighting, flexibly enhancing PE signals for attended features-a core computational principle of predictive coding.

### 2.7 Architectural flexibility: stable one-dimensional computation within multidimensional framework

Having established the modelation within multidimensional framenal prediction errors (PEs), we next demonstrate its capacity for flexible dimensionality reduction. Leveraging the dendritic architecture, the network can revert to one-dimensional PE computation by functionally silencing one feature compartment (implemented by setting the input to −1).

**Figure 7:**
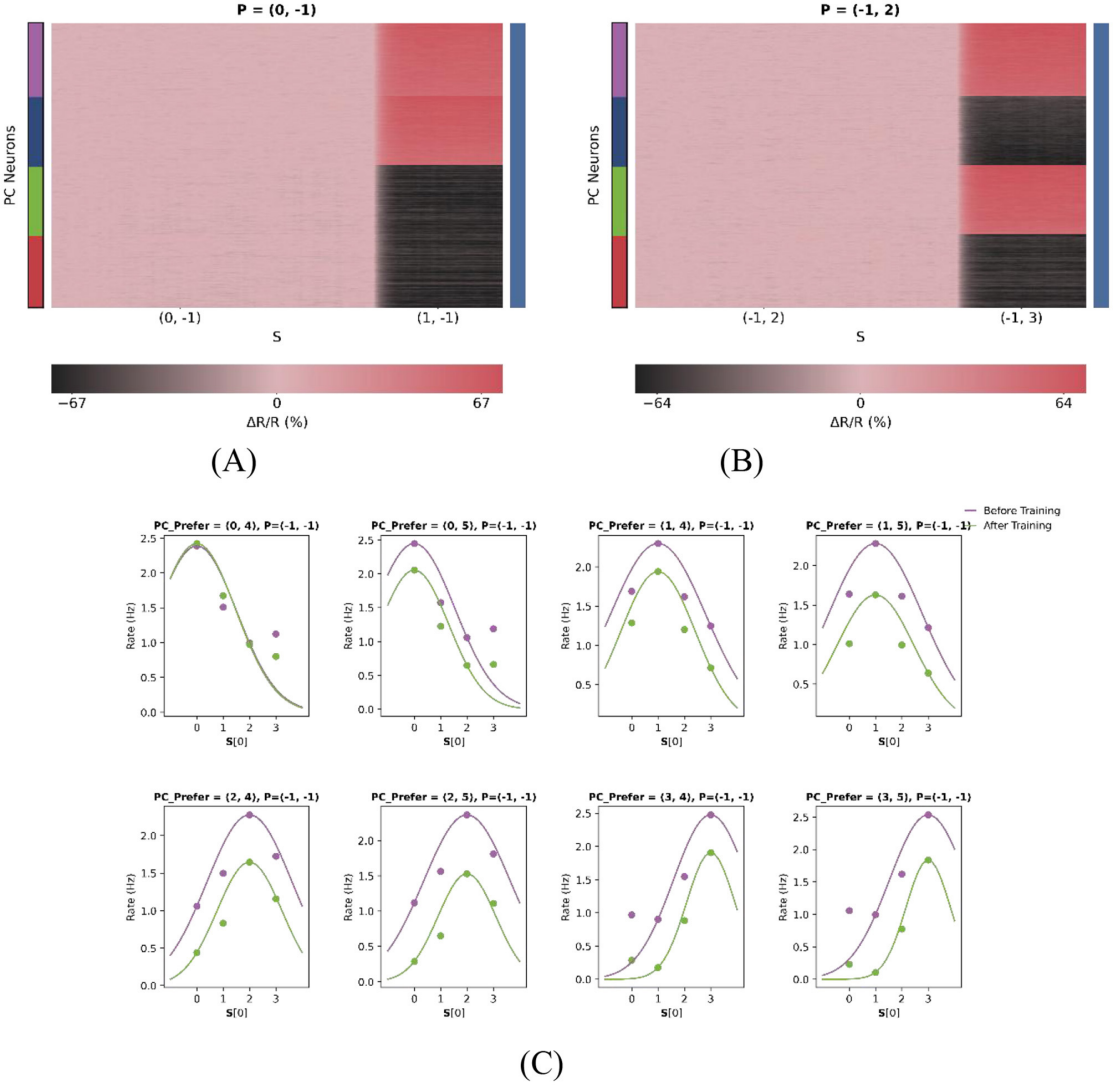
Dimensional reduction preserves core PE properties. **(A**, **B)** Response heatmaps for single-feature input conditions. A value of “−1” denotes the absence of stimulus input in that dimension. **(A)** Feature 1 active (Feature 2 = −1); **(B)** Feature 2 active (Feature 1 = −1). Warmer colors indicate stronger neural responses. **(C)** Stable tuning after dimensional reduction. Each subplot represents one of eight feature-selective neuron types with distinct preferred stimuli. In this configuration, only the first dimension of the two-dimensional stimulus space is considered, as reflected in the x-axis of each subplot. To better fit the Gaussian curve, the number of features was increased from two to four in the first dimension (*F*_1_), with values ranging from 0 to 3, while *F*_2_ was set to 4 and 5. This yielded eight distinct neuron subpopulations, each selectively tuned to a unique stimulus pair. Each subplot shows the population-average responses of PC neurons (scatter points), with Gaussian tuning curves fitted to the data (solid lines). Notably, the emergence of PE neurons after training does not alter their intrinsic tuning, as tuning peaks remain laterally stable.

As illustrated in Figures 7A, B, the model can be effectively reduced to a onedimensional prediction error (PE) neuron model. In this experiment, two test cases were examined. For the first test case *P* = (0, −1) with sensory inputs *S* = (0, −1) and *S* = (1, −1), PE neurons are expected to maintain baseline activity during the (0, −1) type match phase. In contrast, during the (0, −1)−(1, −1) mismatch phase, PE neurons preferring stimuli (1, 2) and (1, 3) - i.e., those with the same first-dimensional feature as the actual stimulus—exhibited relatively pronounced excitation. Similarly, for the second test case *P* = (−1, 2) with sensory inputs *S* = (−1, 2) and *S* = (−1, 3), PE neurons also maintained baseline activity during the (−1, 2) type match phase, while neurons tuned to (0, 3) and (1, 3) stimuli showed stronger responses during the (−1, 2)−(−1, 3) mismatch phase. As shown in Figure 7C, dimensional reduction does not alter the stimulus tuning of the neurons, as their tuning curves do not shift laterally.

By transitioning from a one-dimensional to a two-dimensional model and subsequently reverting to a one-dimensional configuration, we establish a closed-loop validation of the model's robustness. The successful reproduction of the reduced onedimensional PE responses highlights the model's flexibility and generalizability. This supports its applicability in studying sensory prediction error processing across varying levels of complexity, from simple one-dimensional inputs to naturalistic, highdimensional stimuli.

## 3 Discussion

Predictive coding has emerged as a unifying framework for understanding perception and action, positing that the brain continuously generates top-down predictions to suppress incoming sensory signals and updates internal models based on mismatches (termed prediction errors, or PEs) between expected and actual inputs (Friston, 2010; de Lange et al., 2018). A core requirement of this hierarchical inference process is the dynamic coordination of descending predictions and ascending sensory evidence within cortical microcircuits. While prior computational models have demonstrated that inhibitory plasticity can give rise to PE-like responses, it remains unclear how such mechanisms scale to compute feature-specific prediction errors in highdimensional and dynamically changing environments.

**Figure 8:**
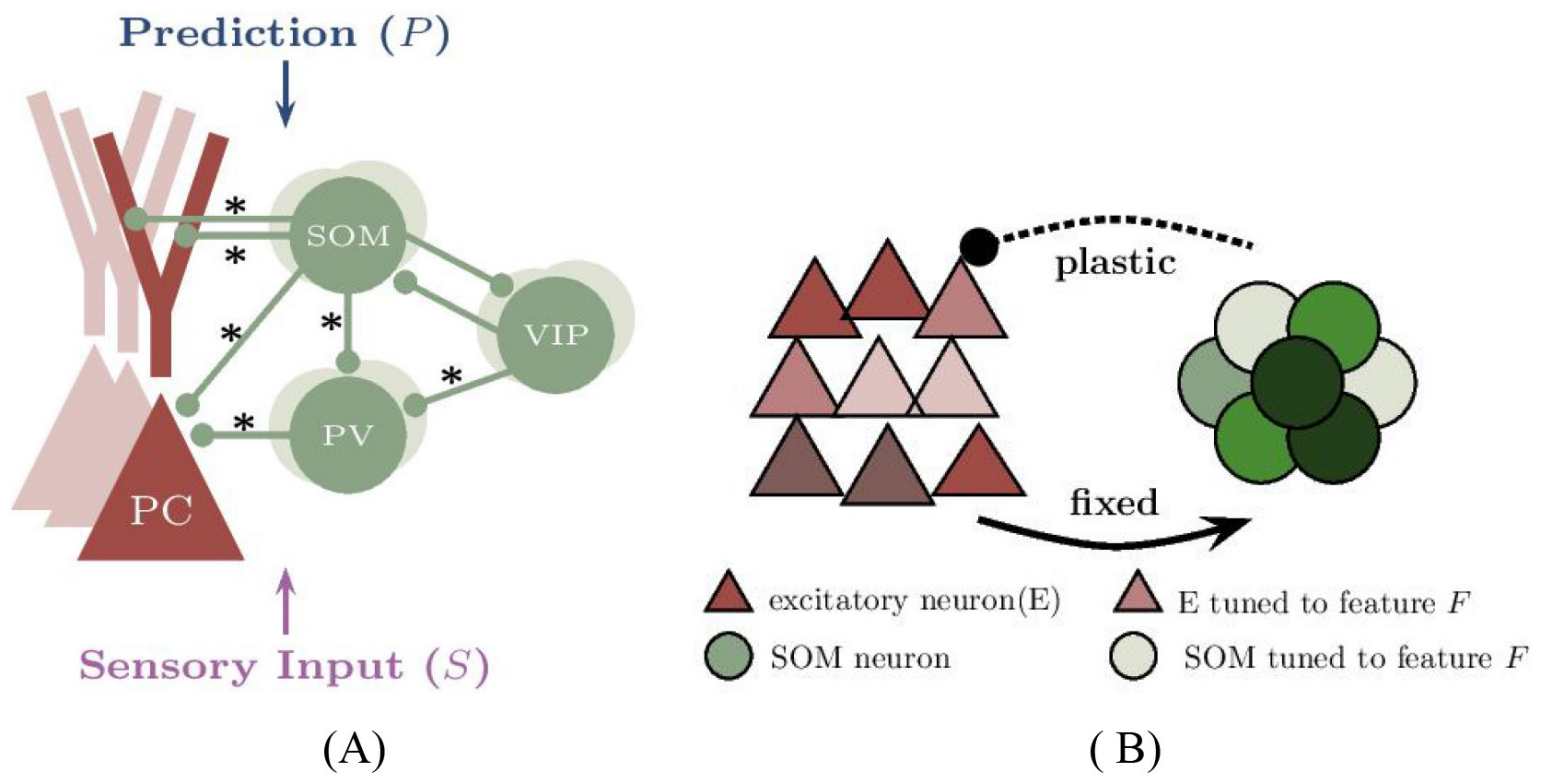
Modeling the neural circuit for one-dimensional prediction error in voluntary behavior. **(A)** The network model comprises excitatory pyramidal cells (PCs) and three types of inhibitory interneurons. The network includes 400 neurons, though connections from PCs are not illustrated for clarity. Connections indicated by an ‘*' are subject to synaptic plasticity. Typically, the somatic regions of PC, SOM, and PV neurons are responsible for receiving visual input (*S*), whereas the dendritic branches of PCs and VIP neurons are involved in receiving motor-related predictions (*P*). Although two dendritic branches are illustrated for compatibility with multidimensional PE modeling, only a single dendritic branch is utilized in the one-dimensional case. **(B)** The subnetwork of excitatory (E) neurons, represented by red triangles, and inhibitory (SOM) neurons, shown as green circles, receives feature-specific tuned input. Excitatory neurons tuned to a sample stimulus “0” are marked in dark red, with their inhibitory counterparts shown in dark green. The network contains four distinct types of selective neurons, each color-coded to reflect their unique stimulus preferences.

In this study, we developed a biologically inspired computational model that integrates the predictive coding framework with experimentally established cortical circuit motifs (Figures 8A, B). These include compartmentalized pyramidal neurons (Yang et al., 2016), three major classes of inhibitory interneurons (PV, SOM, and VIP), and experiencedependent inhibitory plasticity. Our results demonstrate that prediction-driven modulation of pyramidal cell (PC) activity emerges from the tuning of inhibitory interneurons and inhibitory plasticity. The model generalizes to both one- and two-dimensional feature spaces through dendritic compartmentalization, offering a scalable architecture for multidimensional prediction error (PE) computation. This enables the simultaneous processing of multiple features (e.g., orientation and spatial location) while preserving key computational properties of PE circuits: mismatch sensitivity, selective amplification, and content-specific representation.

### 3.1 Feature selectivity in PE computation

Recent computational work on prediction error (PE) neurons, particularly by Hertäg and Sprekeler, 2020 and Hertäg and Clopath, 2022, has advanced our understanding of circuit-level mechanisms in the mouse primary visual cortex (V1). However, these models lack neuron-specific feature selectivity—a fundamental property of sensory cortical circuits. Numerous experimental studies have shown that V1 neurons exhibit strong tuning to specific stimulus features, including orientation, spatial location, and motion direction (Hubel and Wiesel, 1959; Niell and Stryker, 2008). This feature selectivity is not only critical for sensory encoding, but also for generating feature-specific PE signals. Crucially, rodent V1 PE neurons detect retinotopically localized mismatches (Zmarz and Keller, 2016), prioritizing behaviorally relevant deviations over broadcast errors. By leveraging feature selectivity, PE neurons can encode discrepancies along specific stimulus dimensions, supporting context-sensitive and goal-directed behavioral adjustments (Stefanics et al., 2019).

To address the lack of feature selectivity in prior models, we developed a computational framework where both actual and predicted inputs are represented by Gaussian-tuned signals targeting excitatory neurons. These tuning profiles enable the network to differentiate between match and mismatch stimuli along specific dimensions, such as orientation or spatial location. Furthermore, the incorporation of the tuning of inhibitory interneurons (Kerlin et al., 2010), enhances content-specific top-down modulation of cortical activity, contrasting with uniform, non-specific inhibitory schemes. This design aligns with experimental findings from Peelen and Kastner (2011), who demonstrated category-specific preparatory activation in object-selective cortex, even in the absence of bottom-up input. By embedding feature selectivity into both excitatory and inhibitory components of the network, our model supports biologically realistic and computationally efficient prediction error (PE) computation. It demonstrates how feature-selective PE neurons can emerge from structured inputs and plastic interactions, offering a refined understanding of how cortical circuits encode and respond to deviations from sensory expectations.

###  3.2 Model predictions and core contributions

In this study, we developed a computational model to investigate how prediction error (PE) neurons with feature selectivity emerge in cortical circuits through experiencedependent inhibitory plasticity. Our key findings and theoretical contributions can be summarized as follows: (I) Emergence of PE neurons via E/I balancing: our model demonstrates that PE neurons encoding either one-dimensional or two-dimensional stimulus features can arise through synaptic plasticity rules that dynamically balance excitation and inhibition. This form of inhibitory plasticity enables the transformation of initially untuned networks into selective mismatch detectors. (II) Mechanisms of prediction-driven suppression: we show that inhibitory plasticity allows top-down predictions to selectively suppress pyramidal cell (PC) activity. This suppression critically depends on the feature tuning of somatostatin-positive (SOM) interneurons. Without such tuning, the suppression becomes broad and non-specific, underscoring the importance of interneuron selectivity in implementing efficient and selective inhibition. (III) Core properties of PE neurons: PE neurons in our framework exhibit three hallmark properties of predictive coding: (1) Mismatch responses scale with the degree of deviation from prediction; (2) Bottom-up sensory inputs and top-down predictions exert opposing influences, resembling subtractive computation; (3) Mismatch signals are selectively amplified through learning, leading to improved encoding of stimulus feature discrepancies. (IV) Content-specific representation of predictions and errors: in our model, predictions are conveyed through feature-selective input patterns, consistent with evidence that sensory templates can be pre-activated before stimulus onset (Kok et al., 2017). Conversely, prediction errors are encoded as deviations from these expectations, enabling adaptive responses to novel or unexpected events. (V) Attentional modulation of PE responses: our simulations show that when mismatches occur simultaneously across both stimuli dimensions, strong two-dimensional PE responses are generated. Crucially, attention selectively enhances mismatch signals along the attended dimension, consistent with predictive coding accounts of precision-weighting (Friston, 2005; Smout et al., 2019). This suggests a plausible mechanism for flexible and goal-directed modulation of sensory error processing. (VI) Scalable generalization to multidimensional prediction errors: by extending the model from one- to two-dimensional PE circuits, we demonstrate its flexibility and scalability. This generalization only requires the addition of functionally segregated dendritic compartments for each stimulus dimension, while preserving the core architecture of compartmentalized input integration.

Together, these findings provide a comprehensive theoretical account of how cortical circuits may implement prediction error computation in a feature-selective, scalable, and biologically grounded manner. They underscore the essential role of inhibitory plasticity and interneuron tuning in enabling flexible sensory suppression and dynamic error signaling during voluntary behavior.

###  3.3 Learning rules and biological plausibility

A central methodological choice in our model is the use of supervised gradient descent to derive synaptic plasticity rules that enable the emergence of prediction error (PE) neurons. Although this approach diverges from biologically implemented learning mechanisms, it offers a tractable and analytically interpretable framework for identifying the circuit configurations required for accurate PE computation. The resulting rules bear resemblance to biologically inspired homeostatic inhibitory plasticity (Vogels et al., 2011) and error-driven learning algorithms (Rumelhart et al., 1986), serving as a computational abstraction rather than a direct mechanistic implementation. Since the primary objective of this study is to demonstrate that feature-selective PE circuits can be learned through excitation-inhibition (E/I) balancing, we employed gradient descent to derive the plasticity rules to ensure maximal generality.

Crucially, the derived plasticity rules align with experimentally observed principles of inhibitory synaptic plasticity. Specifically, our rule requires inhibitory synapses to adjust their strength based on convergent excitatory inputs to individual pyramidal neurons (Equations 15-17), mirroring empirical findings that inhibitory plasticity is gated by coincident excitatory activity (Liu et al., 2007). This input-specific modulation promotes fine-grained matching of inhibition to excitation at the synaptic level, a mechanism essential for maintaining local E/I balance across neuronal compartments (Tao et al., 2014). Thus, while derived via global optimization, the core logic of our plasticity rules - where inhibition co-varies with excitation - is neurobiologically grounded.

Our gradient-based framework demonstrates that coordinated inhibitory plasticity across compartments enables selective PE responses. This approach systematically explores solution spaces to identify candidate circuit motifs, establishing foundations for future extensions where global optimization is replaced by local mechanisms such as activity-dependent inhibitory plasticity (Vogels et al., 2011; Mackwood et al., 2021) or dendritically compartmentalized learning rules (Bono and Clopath, 2017). Such transitions will enhance biological realism while retaining explanatory power.

###  3.4 Limitations and future directions

While the current model successfully captures key aspects of PE signaling and inhibitory plasticity, it includes several simplifying assumptions. Notably, parvalbuminpositive (PV) interneurons are modeled as receiving actual sensory inputs but lacking feature selectivity. This design choice reflects experimental evidence suggesting that PV interneurons form dense, broadly distributed connections and serve as a source of global inhibition (Packer and Yuste, 2011; Kerlin et al., 2010). However, other studies have reported that PV neurons may exhibit broad but non-uniform feature tuning (Ma et al., 2010; Hofer et al., 2011), which was not implemented in the present model. Future work could examine how incorporating graded PV tuning influences the balance between global and feature-specific inhibition, potentially revealing additional circuit mechanisms that support precision and stability during predictive processing.

Our model focuses on V1 microcircuits but provides a generalizable framework for predictive processing across cortical areas. By predicting complex network behaviors that have yet to be experimentally tested, this work opens new avenues for exploring the context-dependent dynamics of neural networks. In addition to offering mechanistic insights into the generation and modulation of prediction-error signals, our framework establishes a foundation for investigating how such computations support higher-order cognitive functions and behaviors across diverse biological systems.

## 4 Materials and methods

### 4.1 Network model

The neural circuit structure for prediction error is depicted in Figure 8. In the following derivations, we used a two-dimensional prediction error model as an example. If this model is reduced into a one-dimensional scenario, certain terms can be ignored. We simulated a network comprising 280 excitatory pyramidal cells and 120 inhibitory neurons, including PV, SOM, and VIP neurons (N_PV_ = N_SOM_ = N_VIP_ = 40). In the two-dimensional context, the excitatory pyramidal cells (PC) neurons are modeled using a two-compartment model, with the soma, basal dendrites, and axons combined into one compartment and two apical dendritic branches operating independently in another compartment. Inhibitory neurons are modeled using a single-compartment model, referred to as point neurons. Here SOM neurons are further categorized into two subtypes: Martinotti neurons, which are the dominant type and typically connect to the apical dendrites of PC neurons (Rudy et al., 2011; Jiang et al., 2015; Kawaguchi and Kubota, 1997), and non-Martinotti neurons, which may connect to the basal dendrites of PC neurons (McGarry et al., 2010). In our numerical simulations, we adopted a ratio of 7:3 between Martinotti and non-Martinotti neurons to reflect their relative abundance.

1. The pyramidal cells are represented by a two-compartment firing rate model, where E denotes the somatic compartment, and D_1_ and D_2_ represent the two dendritic branches of the pyramidal neurons. The dynamics of the firing rate $r_{i}^{\text{E}}$ of the somatic compartment of the neuron *i* obey

(1) $$\begin{array}{l} \tau_{\text{E}}\frac{\;\text{d}h_{i}^{\text{E}}}{\;\text{d}t}=-h_{i}^{\text{E}}+\left[I_{i}-\Theta \right],\\ \;r_{i}^{\text{E}}={\left[h_{i}^{\text{E}}\right]}_{+}=\max\left\{h_{i}^{\text{E}},0\right\}, \end{array}$$

where τ_*E*_ represents the excitatory rate time constant (τ_*E*_ = 60ms), Θ refers to the rheobase of the neuron (Θ = 14s^−1^) (Hertäg and Sprekeler, 2020). *I*_*i*_ denotes the total somatic input, which includes contributions from somatic and dendritic synaptic activity, as well as potential dendritic calcium spikes.


(2) $$\begin{array}{l} I_{i}=\left(1-\lambda_{\text{E}}\right)I_{\text{E},i}^{\text{syn}}+\lambda_{\text{D}}{\left[I_{{\text{D}}_{1,i}}^{\text{syn}}+c_{i}\right]}_{+}+\lambda_{\text{D}}{\left[I_{{\text{D}}_{2,i}}^{\text{syn}}+c_{i}\right]}_{+}. \end{array}$$

Here, the function [*x*]_+_ = max(*x*, 0) represents a rectifying non-linearity that limits excessive input from the apical dendrite from influencing the soma. $I_{{\text{D}}_{1,i}}^{\text{syn}},I_{{\text{D}}_{2,i}}^{\text{syn}}$, and $I_{\text{E},i}^{\text{syn}}$ denote the total synaptic inputs into two dendritic branches and soma, respectively, and *c*_*i*_ represents a dendritic calcium event. λ_D_ and λ_E_ indicate the fractions of current that leak from the dendrites and soma, with values of λ_D_ = 0.27 and, λ_E_ = 0.31, respectively. Below is a detailed explanation of the specific meanings of each term in the above formula.

(i) $I_{\text{E},i}^{\text{syn}}$ consists of excitatory inputs from outside x^E^, excitatory synaptic inputs from other PC neurons (E), and inhibitory synaptic inputs from PV neurons (P) and nonMartinotti neurons (nM):


(3) $$\begin{array}{l} I_{\text{E},i}^{\text{syn}}={\text{x}}^{\text{E}}+\overset{N_{\text{PC}}}{\underset{j=1,i\neq j}{\sum }}w_{ij}^{\text{EE}}\cdot r_{j}^{\text{E}}-\overset{N_{\text{PV}}}{\underset{j=1}{\sum }}w_{ij}^{\text{EP}}\cdot r_{j}^{\text{P}}-\overset{N_{\text{nM}}}{\underset{j=1}{\sum }}w_{ij}^{\text{EnM}}\cdot r_{j}^{\text{nM}}, \end{array}$$

where the weight matrices $W^{\text{EE}}=\left(w_{ij}^{\text{EE}}\right),W^{\text{EP}}=\left(w_{ij}^{\text{EP}}\right)$, and $W^{\text{EnM}}=\left(w_{ij}^{\text{EnM}}\right)$ denote the synaptic strength from other PC neurons, PV neurons, and non-Martinotti neurons to the PC neuron soma, respectively.

(ii) The dendritic input $I_{{\text{D}}_{k,i}}^{\text{syn}}\left(k=1,2\right)$ consists of excitatory inputs from outside ${\text{x}}^{{\text{D}}_{k}}$, the recurrent connections from other PCs and Martinotti neuron-induced inhibition:


(4) $$\begin{array}{l} I_{{\text{D}}_{k,i}}^{\text{syn}}={\text{x}}^{{\text{D}}_{k}}+\overset{N_{\text{PC}}}{\underset{j=1}{\sum }}w_{ij}^{{\text{D}}_{k}\text{E}}\cdot r_{j}^{\text{E}}-\overset{N_{\text{M}}}{\underset{j=1}{\sum }}w_{ij}^{{\text{D}}_{k}\text{M}}\cdot r_{j}^{\text{M}},k=1,2 \end{array}$$

where the weight matrices $W^{{\text{D}}_{k}\text{E}}=\left(w_{ij}^{{\text{D}}_{k}\text{E}}\right)$ and $W^{{\text{D}}_{k}\text{M}}=\left(w_{ij}^{{\text{D}}_{k}\text{M}}\right)$ denote the recurrence between PCs ( $w_{ij}^{{\text{D}}_{\text{k}}\text{E}}$ ) and Martinotti neurons ( $w_{ij}^{{\text{D}}_{\text{k}}\text{M}}$ ), respectively.

(iii)The input generated by a Ca^2+^ spike is expressed as:


(5) $$\begin{array}{l} c_{i}=c\cdot H\left(I_{{\text{D}}_{k,i}}^{0}-\Theta_{c}\right),k=1,2 \end{array}$$

Here *c* determines the scale of the current produced (*c* = 7*s*^−1^), *H* represents the Heaviside step function, and Θ_*c*_ defines the threshold required to trigger a Ca^2+^-spike $\left(\Theta_{c}=28s^{-1}\right)$. Additionally, $I_{{\text{D}}_{k},i}^{0}$ refers to the total synaptic input generated within the dendrites.


(6) $$\begin{array}{l} I_{{\text{D}}_{k,i}}^{0}=\lambda_{\text{E}}I_{\text{E},i}^{\text{syn}}+\left(1-\lambda_{\text{D}}\right)I_{{\text{D}}_{k,i}}^{\text{syn}},k=1,2 \end{array}$$

2. The firing rate dynamics of each interneuron are modeled by

(7) $$\begin{array}{l} \tau_{\text{I}}\frac{\;{\text{dh}}_{\text{i}}^{\text{Y}}}{\;\text{dt}}=-\;h_{i}^{\text{Y}}+{\text{x}}^{\text{Y}}+\sum_{j=1}^{N_{\text{PC}}}w_{ij}^{\text{YE}}\cdot r_{j}^{\text{E}}-\sum_{j=1}^{N_{\text{PV}}}w_{ij}^{\text{YP}}\cdot r_{j}^{\text{P}}\\ \;-\;\sum_{j=1}^{N_{\text{SOM}}}w_{ij}^{\text{YS}}\cdot r_{j}^{\text{S}}-\sum_{j=1}^{N_{\text{VIP}}}w_{ij}^{\text{YV}}\cdot r_{j}^{\text{V}}\\ \;r_{i}^{\text{I}}={\left[h_{i}^{\text{I}}\right]}_{+}=\max\left\{h_{i}^{\text{I}},0\right\}. \end{array}$$

Here, $h_{i}^{\text{Y}}$ denotes the firing rate of neuron *i* from neuron type Y(Y∈{P, S, V}), and x^Y^ represents the combined external background input and actual or prediction sensory input to Y neurons. The weight matrices $W^{\text{YX}}=\left(w_{ij}^{\text{YX}}\right)$ specifies the connection strength between postsynaptic neuron population Y and presynaptic neuron population X. The firing rate is truncated to ensure non-negativity.

### 4.2 Stimulus selectivity

Simple cells in the primary visual cortex (V1) exhibit selectivity to various stimulus properties, such as color, orientation, motion direction, and location. In our model, we replicated the stimulus tuning observed in pyramidal cells in layer 2/3 of mouse V1 by providing each of the 280 excitatory neurons and 40 SOM neurons with external excitatory input tuned to one- or two-dimensional Gaussian stimuli, consistent with experimental findings (Niell and Stryker, 2008; Sohya et al., 2007). The preferred stimuli of these PC and SOM neurons were evenly distributed across the stimulus space. Within this framework, prediction and actual sensory inputs are represented by stimulus features and prediction errors are evaluated based on the disparities between predicted and observed stimulus features. Notably, the PCs in this network generally exhibit four types of stimulus selectivity either in one or two dimensions, and each selective neuron was assumed to be homogeneous. Therefore, when analyzing the properties of neurons in the network, we considered these neuron types separately.

To develop a neural circuit model for prediction errors from one- or two-dimensional stimuli, it is necessary to define each neuron's feature selectivity, which determines how neurons respond to different stimulus values. This selectivity is typically represented by one- or two-dimensional tuning curves that describe how neurons encode and respond to various stimulus features, such as direction and color in visual neurons. For high-dimensional stimuli, these tuning curves reveal which combinations of stimuli evoke the most robust responses. We used Gaussian functions to approximate the tuning curve of a neuron with feature selectivity. The response of a neuron with a preferred stimulus **s**_max_ to a stimulus **s** can be expressed as:


(8) $$\begin{array}{l} f\left({\text{s}}_{\max},\text{s}\right)=r_{\max}\exp\left(\frac{1}{2}{\left(\text{s}-{\text{s}}_{\max}\right)}^{\text{T}}\overset{-1}{\sum }\left(\text{s}-{\text{s}}_{\max}\right)\right) \end{array}$$

Here, **s**_max_ and **s** are *n*-dimensional vectors, where **s** = [*s*_1_, ⋯ , *s*_*n*_] and *n* is the number of stimulus features. Each component represents a value in a specific stimulus dimension (feature) and **s**_max_ denotes the *n*-dimensional preferred stimulus of the neuron. Both vectors share the same range of values. Σ denotes the covariance matrix, which is symmetric and positive definite.


$$\begin{array}{l} \Sigma =\left(\begin{array}{cccc} \sigma_{11} & \sigma_{12} & \cdots & \sigma_{1k}\\ \sigma_{21} & \sigma_{22} & \cdots & \sigma_{2k}\\ \vdots & \vdots & \ddots & \vdots \\ \sigma_{k1} & \sigma_{k2} & \cdots & \sigma_{kk} \end{array}\right), \end{array}$$

where σ_*ij*_ represents the covariance between the *i*-th and *j*-th fetaure. If the different features of the stimulus are uncorrelated, this matrix becomes a diagonal matrix.

In this study, we considered the different stimulus features to be independent, so the off-diagonal elements σ_*ij*_ are set to zero. Additionally, we assumed that all diagonal elements σ_*ii*_ had the same value. Therefore, for two-dimensional tuning curves, we have the following formula:


(9) $$f\left(s_{\max},s\right)=r_{\max}e^{-\frac{1}{2\sigma^{2}}[{\left(s[0]-s_{\max}[0]\right]}^{2}+\left(s[1]-s_{\max}{\left[1\right)}^{2}\right]},$$

for one-dimensional PE neurons, this expression reduces to a one-dimensional Gaussian tuning function.

To simulate selective attention, we increased the sensitivity of neurons to the attended dimension by amplifying the difference term of the attended feature before computing the response. Specifically, during testing, when attention was directed to feature 1 (first dimension), the difference **s**[0]−**s**_max_[0] was scaled by a factor of 1.2 prior to computing the response, while feature 2 remained unchanged. Conversely, when attention was directed to feature 2, the term **s**[1]−**s**_max_[1] was scaled by 1.2. This manipulation effectively narrowed the tuning bandwidth for the attended dimension, simulating an increase in precision without altering the original connectivity or learning rule.

Our study considered the sensory stimulus F with a value range denoted by *F*. Specifically, focusing on the orientation feature in selective neurons, the preferred directions F of the neurons have a range of {0°, 30°, 60°, 90°}. Initially, our analysis focused on one-dimensional stimuli, where the range of *F* was typically limited to {0, 1, 2, 3}. To compare this model with experimental results, these values can be mapped to real-world values, and parameters adjusted, none affecting the model's conclusions.

For two-dimensional stimuli, the value space for the first-dimensional feature *F*_1_ is restricted to {0, 1}, while the second feature *F*_2_, is limited to {2, 3}. Consequently, there are four types of stimulus selectivity in the network, *F* = {(0, 2), (0, 3), (1, 2), (1, 3)}.

### 4.3 PE neurons

The feedforward sensory input is denoted by *S*, and the feedback from the upper level cortical regions is denoted by *P*. When neither *P* nor *S* is present, we refer to this state as the baseline state (BL). In the match phase, when *P* = *S* = **s**_0_∈*F*, it is called the **s**_0_-type match phase. Conversely, when *P* = **s**_1_≠**s**_2_ = *S*∈*F*, we refer to it as the **s**_1_−**s**_2_ type mismatch phase.

In the prediction error circuit, top-down predictions originating from higher cortical areas are thought to inhibit the activity of excitatory neurons in a feature-specific manner (Fuehrer et al., 2022). This inhibitory effect is stronger for PC neurons whose tuning matches the predicted features. Therefore, prediction error (PE) neurons are expected to exhibit the following properties:

(i) Neuronal activity remains at baseline during the *P*-type match phase;

(ii) The neurons with preferred stimulus **s**_max_ show the strongest activity at **s**_0_−**s**_max_ type mismatch phase and maintain baseline or slight suppression at **s**_max_−**s**_0_ type mismatch phase;

(iii) In the **s**_1_−**s**_2_ type mismatch phase, neuron activity is inversely proportional to the similarity between **s**_1_ and the neuron's preferred stimulus and directly proportional to the similarity between **s**_2_ and the preferred stimulus.

The activity of a prediction error (PE) neuron indirectly reflects the similarity between predicted and preferred stimulus values, enabling effective differentiation between stimuli and providing valuable feedback. For example, if a PE neuron prefers the value 1, it responds most strongly to a ‘0 − 1' mismatch and maintains baseline activity for an expected stimulus.

### 4.4 Selective suppression

Given a neuron with a preferred stimulus **s**_max_ and an input stimulus **s**_0_, **s**_max_, **s**_0_∈*F*, the similarity between **s**_0_ and the selectivity **s**_max_ of the neuron for the two-dimensional feature is defined as follows:


(10) $$\begin{array}{l} RS\left({\text{s}}_{\max},{\text{s}}_{0}\right)=1-\mu \left(\frac{{\text{s}}_{\max}-{\text{s}}_{0}}{\left||{\text{s}}_{\max}||+||{\text{s}}_{0}|\right|}\right), \end{array}$$

where $\mu \left(\text{s}\right)=\frac{1}{n}\overset{n}{\underset{i=1}{\sum }}|s_{i}|$. And for one-dimensional feature, we define the similarity as follows:


(11) $$\begin{array}{l} RS\left(s_{\max},s_{0}\right)=\frac{f\left(s_{\max},s_{0}\right)}{f_{\max}} \end{array}$$

where *f*(·, ·) is the tuning curve of the neuron and *f*_max_ is its maximum.

To measure the strength of selectivity, the selectivity coefficient of *P* for PC neurons is defined as:


(12) $$\begin{array}{l} \gamma \left(P\right)=\frac{{\langle {\left(\langle r_{s}^{\text{E}}\rangle -{\bar{r}}^{\text{E}}\right)}^{3}\rangle }_{s}}{{\langle {\left(\langle r_{s}^{\text{E}}\rangle -{\bar{r}}^{\text{E}}\right)}^{2}\rangle }_{s}^{3/2}} \end{array}$$

Here, 〈·〉_*s*_ denotes averaging across PC neurons with different selectivities. $\langle r_{s}^{\text{E}}\rangle$ represents the average steady-state firing rate (averaged over time and population) of the PC population with the preferred stimulus *s* in the presence of the only stimulus *P* and ${\bar{r}}^{\text{E}}$ is the average population response. This equation is adapted, with slight modifications, from the method proposed by Znamenskiy et al. (2024). The greater the difference in response to stimulus *P* among PC neurons with different selectivities, the stronger the selective suppression. A large absolute value of |γ(*P*)| indicates that feature-selective suppression across PC neurons is strong. We empirically define the threshold of significance as |γ(*P*)|>0.5. Given sufficiently clear selectivity, the greater the activity reduction of PC neurons whose preferred stimulus is *P* below baseline, the stronger the selective suppression is considered to be.

### 4.5 Excitatory and inhibitory pathways of PC neurons

We aggregated all learned synaptic weights after training to construct the weight matrix **W**, which is organized based on the shared stimulus preference of each homogeneous population using the formulation provided in Equation 3 of the Supplementary material. Initially, **W** included only connections to the somatic compartments of PC neurons, excluding dendritic components. To incorporate excitatory and inhibitory contributions from dendrites, **W** was expanded by adding 2*n* rows and 2*n* columns representing dendritic elements, including recurrent and inhibitory weights originating from SOM neurons. Here, *n* denotes the number of PC neuron subpopulations with distinct feature preferences in the network. By computing the inverse of **W**, denoted as **M**, we could calculate the steady-state firing rate as: **r** = −**M**·**S**, where **S** is a vector composed of prediction input and actual sensory input delivered to the network (including background stimuli). From **M**, the first *n* rows were extracted to obtain **M**_soma_ and the subsequent 2*n* rows to obtain **M**_dend1_ and **M**_dend2_, corresponding to the excitatory and inhibitory pathways targeting the PC soma and dendritic compartments, respectively.

Due to the complex interactions within the network, the sign of **M**_*ij*_ is not solely determined by the corresponding synaptic weight *w*_*ij*_. Therefore, the net excitatory and inhibitory inputs to PC neuron soma and dendrites can be computed as follows:


$$\begin{array}{l} {\text{pathSoma }}_{E}=\left(M_{\text{soma }}{}^{\circ}\Gamma_{M_{\text{soma }}>0}\right)\cdot \text{S}[1:\text{n}],\;\\ {\text{pathSoma }}_{I}=\left|M_{\text{soma }}{}^{\circ}\Gamma_{M_{\text{soma }}<0}\right|\cdot \text{S}[1:\text{n}],\;\\ {\text{pathDend1 }}_{E}=\left(M_{\text{dend1 }}{}^{\circ}\Gamma_{M_{\text{dend1 }}>0}\right)\cdot \text{S}[\text{n}:2\text{n}],\;\\ {\text{pathDend1 }}_{I}=\left|M_{\text{dend1 }}{}^{\circ}\Gamma_{M_{\text{dend1 }}<0}\right|\cdot \text{S}[\text{n}:2\text{n}],\;\\ {\text{pathDend2 }}_{E}=\left(M_{\text{dend2 }}{}^{\circ}\Gamma_{M_{\text{dend2 }}>0}\right)\cdot \text{S}[2\text{n}:3\text{n}],\;\\ {\text{pathDend2 }}_{I}=\left|M_{\text{dend2 }}{}^{\circ}\Gamma_{M_{\text{dend2 }}<0}\right|\cdot \text{S}[2\text{n}:3\text{n}], \end{array}$$

where ° denotes element-wise (Hadamard) multiplication. Γ_*A*>0_ is an indicator matrix of the same size as *A*, with entries 1 where *A*_*ij*_>0 and 0 otherwise. S[1:n], S[n:2n], and S[2n:3n] represent the corresponding partitions of the input vector.

**Figure 9:**
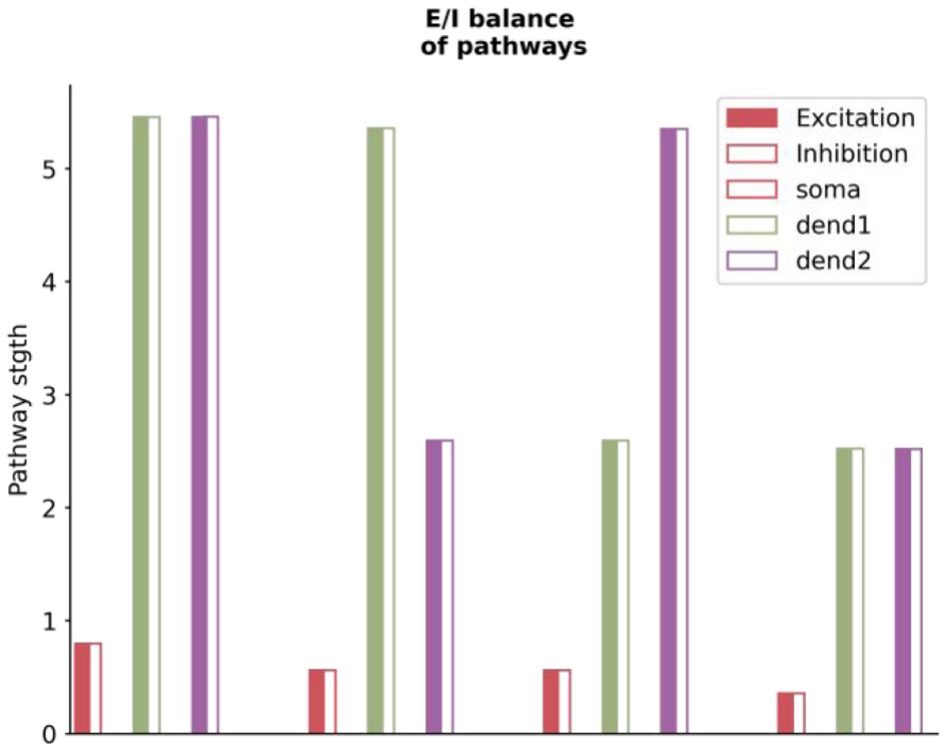
The excitatory and inhibitory balance in pathways to stimulus-selective neurons across different compartments. The x-axis depicts various neuron compartments, including soma and two dendritic branches, corresponding to each type of stimulus-selective neuron. The y-axis represents the intensity of excitatory or inhibitory projections, with solid bars indicating excitatory inputs and hollow bars representing inhibitory inputs. The balance of excitatory and inhibitory pathways is critical for maintaining the baseline activity of PE neurons, regardless of stimulus strength. The *S* used in the graph is the match stimulus, i.e., *P* = *S* = (0, 2). The plotted values are derived using the analytically computed weight matrix *W* from the Supplementary material.

Our analysis reveals that the balance of excitatory and inhibitory (E/I) inputs to prediction error (PE) neurons extends beyond just the total input received. It also involves the specific pathways these inputs take within the circuit (see Figure 9). To illustrate this, we calculate the cumulative effect of all pathways originating from specific neuron types or compartments, terminating at either the soma or dendrites of PE neurons. These contributions, categorized as net excitatory or inhibitory, highlight an inherent E/I balance. This equilibrium ensures that PE neurons can maintain baseline activity regardless of changes in stimulus strength.

### 4.6 Random network model

Assuming that neurons of the same type share an equal number of afferent connections, the directed connection probabilities between any two neuron types can be determined based on the synaptic type (Packer and Yuste, 2011; Fino and Yuste, 2011; Jiang et al., 2015).


(13) $$\begin{array}{l} \text{P}=\left(\begin{array}{lllll} p^{\text{EE}} & p^{\text{ED}} & p^{\text{EP}} & p^{\text{ES}} & p^{\text{EV}}\\ p^{\text{DE}} & p^{\text{DD}} & p^{\text{DP}} & p^{\text{DS}} & p^{\text{DV}}\\ p^{\text{PE}} & p^{\text{PD}} & p^{\text{PP}} & p^{\text{PS}} & p^{\text{PV}}\\ p^{\text{SE}} & p^{\text{SD}} & p^{\text{SP}} & p^{\text{SS}} & p^{\text{SV}}\\ p^{\text{VE}} & p^{\text{VD}} & p^{\text{VP}} & p^{\text{VS}} & p^{\text{VV}} \end{array}\right)\\ \;=\left(\begin{array}{ccccc} 0.8 & 1 & 0.6 & 0.54 & 0\\ 0.1 & 0 & 0 & 0.55 & 0\\ 0.45 & 0 & 0.5 & 0.6 & 0.5\\ 0.35 & 0 & 0 & 0 & 0.5\\ 0.1 & 0 & 0 & 0.45 & 0 \end{array}\right), \end{array}$$

where *p*^*XY*^ denotes the probability of a synaptic connection between neurons of type *X* and type *Y*. Similarly, we can define the average total connection strength of a type of neuron that receives a specific type of synapse:


(14) $$\begin{array}{l} \bar{W}=\left(\begin{array}{ccccc} {\bar{w}}^{\text{EE}} & {\bar{w}}^{\text{ED}} & {\bar{w}}^{\text{EP}} & {\bar{w}}^{\text{ES}} & {\bar{w}}^{\text{EV}}\\ {\bar{w}}^{\text{DE}} & {\bar{w}}^{\text{DD}} & {\bar{w}}^{\text{DP}} & {\bar{w}}^{\text{DS}} & {\bar{w}}^{\text{DV}}\\ {\bar{w}}^{\text{PE}} & {\bar{w}}^{\text{PD}} & {\bar{w}}^{\text{PP}} & {\bar{w}}^{\text{PS}} & {\bar{w}}^{\text{PV}}\\ {\bar{w}}^{\text{SE}} & {\bar{w}}^{\text{SD}} & {\bar{w}}^{\text{SP}} & {\bar{w}}^{\text{SS}} & {\bar{w}}^{\text{SV}}\\ {\bar{w}}^{\text{VE}} & {\bar{w}}^{\text{VD}} & {\bar{w}}^{\text{VP}} & {\bar{w}}^{\text{VS}} & {\bar{w}}^{\text{VV}} \end{array}\right)\\ \;=\left(\begin{array}{ccccc} 0.42 & - & 1.75^{*} & 0.35^{*} & -\\ 0.42 & - & - & 0.35^{*} & -\\ 2.5 & - & 0.5 & 0.3^{*} & 0.6^{*}\\ 1 & - & - & - & 0.6\\ 1 & - & - & 0.5 & - \end{array}\right), \end{array}$$

where ${\bar{w}}^{\text{XY}}$ represents the average input from population *Y* to a unit in population *X*. The symbol ‘*' indicates that the weight of these synapses is plastic and can adapt to optimize network behavior, whereas ‘-' indicates the absence of a synaptic connection between these neuron types. Specifically, each unit in population *X* receives, on average, $p^{XY}N_{Y}$ projections from population *Y*, where *N*_*Y*_ is the number of units in population *Y*. The synaptic connections are initialized based on $\bar{W}$, with $w_{ij}^{\text{initial}}\in U\left(0.5\bar{w},1.5\bar{w}\right)/N_{w}$, where *U*(·, ·) represents a uniform distribution and *N*_*w*_ corresponds to the number of such synapses per postsynaptic neuron. Generally, *w*^EP^, *w*^ES^, *w*^DS^, *w*^PS^, and *w*^PV^ are updated by learning rules we derived (Equations 15-17), with learning rates η^EP^ = 10^−4^, η^ES^ = 10^−7^, η^DS^ = 10^−6^, η^PS^ = 10^−6^, and η^PV^ = 10^−6^, respectively. Here, D denotes both D_1_ and D_2_.

To demonstrate the robustness of our results and show that they are not specific to certain connectivity matrices, we apply perturbations to these matrices by scaling each entry with a random variable uniformly distributed within a predefined range. This process randomly adjusts each connection by a small fraction of its original value.

### 4.7 Input

To maintain a reasonable baseline firing rate when there is no sensory input, all neurons receive a constant background input represented by the symbol *x*. In networks, different types of neurons are assigned specific values for their external background input: *x*^E^ = 28Hz, *x*^P^ = *x*^S^ = *x*^V^ = 2Hz, and *x*^D^ = 0Hz. These input values are chosen to ensure that the baseline firing rates of PC neurons are maintained at ρ_E_ = 1.25 Hz.

The visual and motor inputs to excitatory neurons are modeled using bell-shaped tuning curves over the stimulus space, following the approach of Znamenskiy et al. (2024). These inputs are implemented as Gaussian functions with a maximum firing rate of 30 Hz and a tuning width of 0.8. Similarly, the selectivity of SOM neurons is modeled with Gaussian functions, but with a broader tuning width of 1, based on evidence that GABAergic neurons are generally less selective to stimulus orientation than excitatory neurons (Sohya et al., 2007). To validate the robustness of the model against variations in inhibitory selectivity, we conduct a control experiment in which the tuning width of SOM neurons is set equal to that of pyramidal cells (i.e., σ_PC_ = σ_SOM_ = 1 ) (see Supplementary Figure 2).

The 280 excitatory cells have preferred stimuli that are evenly distributed across the stimulus space: 0, 1, 2, 3 in one-dimensional situations, and (0, 2), (0, 3), (1, 2), (1, 3) in two-dimensional situations. All simulations focus only on cases where, whenever the prediction input *P* is present, it is fixed at (0, 2) in the two-dimensional case and at 0 in the one-dimensional case. During training, when sensory input is present, *S* is fixed at *S* = (0, 2) (*S* = 0 in one-dimensional case), indicating that PC neuron activity is trained to remain the baseline firing rate during the (0, 2)-type match phase ( 0 -type match phase in one-dimensional case). The absence of *P* and *S* is denoted by *P* = (−1, −1) and *S* = (−1, −1), respectively ( *P* = −1 and *S* = −1 in one-dimensional case). All training and testing stimuli include random normal perturbations with a mean of 0 and a variance of σ_noise_ = 0.35.

### 4.8 Plasticity

In our model, prediction error (PE) neurons arise via inhibitory plasticity mechanisms that establish excitation-inhibition (E/I) balance in pyramidal cells (PCs). To derive the corresponding inhibitory plasticity rules, we employ gradient descent as a theoretical framework aimed at minimizing the prediction error. Following the approach proposed by Hertäg and Sprekeler (2020), we constrain synaptic plasticity to five inhibitory connections in the network: *w*^EP^, *w*^ES^, *w*^DS^, *w*^PS^, *w*^PV^. These include inhibitory projections from PV and SOM interneurons onto both the somatic and apical dendritic compartments of PCs, as well as inhibitory connections from SOM and VIP neurons onto PV interneurons. To ensure non-negativity of all synaptic weights during the derivation process, we reparameterize the weights as *w* = *s*^+^(*v*), and treat *v* as the optimization variable in gradient descent for minimizing the error function.

The learning rules we derive (see Supplementary material for derivation) are given as follows:


(15) $$\begin{array}{lll} \Delta v_{ij}^{\text{E}X} & = & \eta^{\text{E}X}\left(r_{i}^{\text{E}}-\rho_{i}\right)\frac{\partial W^{\text{E}X}}{\partial v_{ij}^{\text{E}X}}r_{j}^{X},X\in \left\{\text{P},\text{S}\right\}, \end{array}$$

(16) $$\begin{array}{lll} \Delta v_{ij}^{{\text{D}}_{\text{k}}\text{S}} & = & \eta^{D_{k}\text{S}}\left(\lambda_{\text{D}}\left(r_{i}^{\text{E}}-\rho_{i}\right)+\left(A_{i}^{{\text{D}}_{k}}-\epsilon \right)\right)\frac{\partial W^{{\text{D}}_{k}\text{S}}}{\partial v_{ij}^{{\text{D}}_{k}\text{S}}}r_{j}^{\text{S}},\;k=1,2, \end{array}$$

where ρ_*i*_ denotes the target (baseline) firing rate of PCs. In the two-dimensional case, each PC has two dendritic branches, and thus Equation 16 is instantiated separately for each branch. In contrast, in the one-dimensional case, each PC neuron contains a single dendritic branch, and therefore Equation 16 reduces to a single instance. Additionally, $A_{i}^{{\text{D}}_{k}}$ represents the activity of the *k*-th dendritic compartment and ϵ is computed as the rectified sum of synaptic events received by that compartment. Specifically, it is defined as:


$$\begin{array}{l} A_{i}^{{\text{D}}_{k}}=I_{{\text{D}}_{k,i}}^{\text{syn}}+c_{i},k=1,2 \end{array}$$

As shown in Equations 15, 16, the synapses onto both the somatic and dendritic compartments of PCs follow an inhibitory plasticity rule similar to that proposed by Vogels et al. (2011). These rules adjust inhibitory synaptic weights in proportion to the presynaptic interneuron activity and the deviation of postsynaptic PC activity from its baseline level.

Furthermore, for the connections from SOM and VIP neurons onto PV interneurons, we derive the following plasticity rule:


(17) $$\begin{array}{l} \Delta v_{ij}^{\text{PY}}=-\eta^{\text{PY}}\left[\overset{N_{\text{PC}}}{\underset{k=1}{\sum }}\left(r_{k}^{\text{E}}-\rho_{k}\right)W_{ki}^{\text{EP}}\right]\frac{\partial W^{\text{PY}}}{\partial v_{ij}^{\text{PY}}}r_{j}^{\text{Y}},Y\in \left\{\text{S},\text{V}\right\}. \end{array}$$

This rule changes the synapses onto PV neurons in proportion to the presynaptic interneuron activity and the average deviation of the postsynaptic PCs from their baseline rate, following an approximated backpropagation-of-error rule akin to that proposed by Rumelhart et al. (1986).

All synaptic weights are updated after the network reaches a steady-state firing rate in response to each input. The corresponding learning rates η^EP^, η^ES^, η^DS^, η^PS^ are provided in the *Simulations* section.

## Data Availability

The original contributions presented in the study are included in the article/Supplementary material, further inquiries can be directed to the corresponding author.
