# Supplementary material for: Circuit-level modeling of prediction error computation of multi-dimensional features in voluntary actions
Source: Front Comput Neurosci. 2025 Sep 29;19:1551555. doi: 10.3389/fncom.2025.1551555 (PMC12515960; doi:10.3389/fncom.2025.1551555)
Supplement: Supplementary file 1 [file Data_Sheet_1.docx]

Supplementary Material

## 1. Simplified Model

In this study, we examine a simplified model to determine the constraints imposed on the interneuron circuit by the presence of selectively tuned PE neurons. Our derivation, which generalizes to $n$ populations of PCs with distinct feature selectivity, is based on the following key assumptions and considerations (Hertäg and Clopath, 2022, Mackwood et al., 2021 ):

1. PC neurons sharing the same preferred stimulus can be treated as homogeneous populations;
2. The following analysis only considers the case where SOM neurons have no selectivity. A similar analysis can be conducted when SOM neurons exhibit selectivity;
3. At baseline and match phase, the inhibition produced by SOM neurons is not less than the excitatory input reaching the apical dendrites of PC neurons, and all types of neurons have positive firing rate, making the nonlinear truncation of $h^{X}$ negligible (i.e., $r^{X}=h^{X}$).
4. Synaptic connections within the circuit are assumed to be sufficiently weak such that higher-order correlations between synaptic weights can be ignored.
5. Feature independence: different stimulus features are assumed to be statistically independent, allowing separable analysis across feature dimensions.

Under the previous assumptions about the network above, we can simplify the model by neglecting the dendritic compartment of PCs and related synapses. As a result, we can reduce the high-dimensional system to $n+4$ equations (Hertäg and Clopath, 2022). Each equation describes the dynamics of one representative firing rate per neuron type:

$\tau\frac{\text{d}\mathbf{r}}{\text{d}t}=-\mathbf{r}+\Omega\mathbf{r}+\mathbf{X}\text{,}$ (1)

where $\Omega$ is the weight matrix, and $\mathbf{X}$ is the network external input minus the neuron threshold. The external input includes background input and either feedforward sensory input or feedback input.

$\text{r}=\left[\begin{matrix} r^{\text{E}_{1}} \\ \vdots\\ r^{\text{E}_{n}} \\ \mathbf{r}^{\mathbf{I}} \end{matrix}\right],\text{X}=\left[\begin{matrix} \text{x}^{\text{E}_{1}}-\Theta\\ \vdots\\ \text{x}^{\text{E}_{n}}-\Theta\\ \mathbf{X}^{I} \end{matrix}\right],$ (2)

where $r^{\text{E}_{i}}$ is the representative population firing rate of the somatic compartment of the PC neuron population with $i$-th preferred stimulus. $\mathbf{r}^{\text{I}}=[r^{\text{P}},r^{\text{M}},r^{\text{nM}},r^{\text{V}}]^{T}$and $\mathbf{X}^{I}=[\text{x}^{\text{P}},\text{x}^{\text{M}},\text{x}^{\text{nM}},\text{x}^{\text{V}}]^{T}$respectively denote the representative firing rates and external input stimuli for populations of PV, M-type SOM (Martinotti neurons), nM-type SOM (non-Martinotti neurons), and VIP neurons. $\Theta$ is the neuron threshold. In the steady state, the firing rates are as follows:

(3)

where represents the identity matrix and

$\mathbf{W}=\left[\begin{matrix} -\frac{1}{1-\lambda_{\text{E}}} & w^{\text{E}_{1}\text{E}_{2}} & w^{\text{E}_{1}\text{E}_{3}} & \cdots& w^{\text{E}_{1}\text{E}_{n}} & -\mathbf{V}^{\text{E}_{1}\mathbf{I}} \\ w^{\text{E}_{2}\text{E}_{1}} & -\frac{1}{1-\lambda_{\text{E}}} & w^{\text{E}_{2}\text{E}_{3}} & \cdots& w^{\text{E}_{2}\text{E}_{n}} & -\mathbf{V}^{\text{E}_{2}\mathbf{I}} \\ \vdots& \vdots& \vdots& \vdots& \vdots& \vdots\\ w^{\text{E}_{n}\text{E}_{1}} & w^{\text{E}_{n}\text{E}_{2}} & w^{\text{E}_{n}\text{E}_{3}} & \cdots& -\frac{1}{1-\lambda_{\text{E}}} & -\mathbf{V}^{\text{E}_{n}\mathbf{I}} \\ \mathbf{V}^{\mathbf{I}\text{E}_{1}} & \mathbf{V}^{\mathbf{I}\text{E}_{2}} & \mathbf{V}^{\mathbf{I}\text{E}_{3}} & \cdots& \mathbf{V}^{\mathbf{I}\text{E}_{n}} & -\mathbf{W}^{\mathbf{II}}-\text{I}_{4} \end{matrix}\right],$ (4)

where $\mathbf{V}^{\mathbf{I}\text{E}i}$ is a column vector of length $N_{\mathbf{I}}=4$, representing the synaptic weights from the PC neuron with the $i$-th preferred stimulus to all interneurons. Similarly, $\mathbf{V}^{\text{E}_{i}\mathbf{I}}$ is a row vector of the same dimension as $\mathbf{V}^{\mathbf{I}\text{E}_{i}}$, containing the synaptic weights from all interneurons to the PC neuron with the $i$-th preferred stimulus. Additionally, $\mathbf{W}^{\mathbf{II}}$represents effective connections between the four interneuron populations. Equation (3) can be written as

$-\mathbf{Wr}=\mathbf{X}.$ (5)

The equation yields

$\left\{\begin{matrix} \frac{r^{\text{E}_{i}}}{1-\lambda_{\text{E}}}=\text{x}^{\text{E}_{i}}+\sum_{j\neq i}^{n} w^{\text{E}_{i}\text{E}_{j}}r^{\text{E}_{j}}-\mathbf{V}^{\text{E}_{i}\mathbf{I}}r^{\mathbf{I}}-\Theta,i=1,2\cdots n, \\ \mathbf{r}^{\text{I}}=(\mathbf{W}^{\text{II}}+\mathbf{I}_{4})^{-1}(\mathbf{X}^{\mathbf{I}}+\sum_{j=1}^{n} \mathbf{V}^{\mathbf{I}\text{E}_{j}}r^{\text{E}_{j}}). \end{matrix}\right.$ (6)

In the theoretical network model, to selectively inhibit PC neurons by prediction, various PC neuron population activities are trained to satisfy the condition in $P$-type match phase :

$r_{\text{m}}^{\text{E}_{i}}=\rho_{i}$ (7)

where $i=1,2,\cdots,n$. $\rho_{i}$ is the baseline rate of the PC neuron population with $i$-th preferred stimulus.

The neural activity in the $P$-type match phase satisfies the linear system, which can be obtained by considering the Equation ([6](#eq:constrainE)).

$\begin{matrix} \frac{r_{\text{BL}}^{\text{E}_{i}}}{1-\lambda_{\text{E}}}=\text{x}^{\text{E}_{i}}+\sum_{j\neq i}^{n} w^{\text{E}_{i}\text{E}_{j}}\rho_{j}-\mathbf{V}^{\text{E}_{i}\mathbf{I}}\cdot\left(\mathbf{W}^{\mathbf{II}}+\mathbf{I}_{4}\right)^{-1}\cdot\left(\mathbf{X}^{\mathbf{I}}+\sum_{j=1}^{n} \mathbf{V}^{\mathbf{I}\text{E}_{j}}r_{\text{BL}}^{\text{E}_{j}}\right)-\Theta\\ \text{ }=:T(\mathbf{w}). \end{matrix}$ (8)

Note that $\mathbf{w}=[\{w^{\text{E}_{i}\text{P}}\}_{i=1}^{n},\{w^{\text{E}_{i}\text{nM}}\}_{i=1}^{n},w^{\text{PS}},w^{\text{PV}}]$. To find these weight values that satisfy the above equation, we must solve an optimization problem as follows:

$\begin{matrix} \mathop{\text{min}}\limits_{\text{w}} \sum_{i=1}^{n} \left(\frac{\rho_{i}}{1-\lambda_{\text{E}}}-T(\mathbf{w})\right)^{2}. \end{matrix}$ (9)

The solution to the optimization problem yields the theoretical weight values $\{w^{\text{E}_{i}\text{P}}\}_{i=1}^{n}$, $\{w^{\text{E}_{i}\text{nM}}\}_{i=1}^{n}$, $w^{\text{PS}}$, and $w^{\text{PV}}$. In [Equation (8](#eq:constrainE)), there are no constraints on $\{w^{\text{D}_{1,i}\text{M}}\}_{i=1}^{n}$ and $\{w^{\text{D}_{2,i}\text{M}}\}_{i=1}^{n}$. As it is assumed that the dendritic activity of the PC neuron in the match phase is maintained $\varepsilon$ near the baseline of the dendrite, these weight values need to be calculated separately. Combined [Equation (4](#eq:constrainE)) and [Equation (7](#eq:constrainE)) (In the Materials and Methods section),

$\begin{matrix} I_{\text{D}_{k,i}}^{\text{syn}}+c_{i}=\text{x}^{\text{D}_{k,i}}+\sum_{j=1}^{n} w^{\text{D}_{k,i}\text{E}_{j}}\rho_{j}-w^{\text{D}_{k,i}\text{M}}r^{\text{M}}+c_{i},\text{ k=1,2, }i=1,2,\cdots,n. \end{matrix}$ (10)

and

$\tau_{\text{I}}\frac{\text{d}r^{X}}{\text{d}t}=-r^{X}+\text{x}^{X}+\sum_{i=1}^{n} w^{X\text{E}_{i}}\rho_{i}-w^{X\text{P}}r^{\text{P}}-w^{X\text{M}}r^{\text{M}}-w^{X\text{nM}}r^{\text{nM}}-w^{X\text{V}}r^{\text{V}},\text{ }X=\text{M},\text{nM},\text{V}.$ (11)

Since the activity of dendrites of PC neurons in the match phase is close to 0, that is $I_{D_{k,i}}^{syn}+c_{i}\approx0$, we obtain

$w^{\text{D}_{1,i}\text{M}}\approx\frac{\text{x}^{\text{D}_{1,i}}+\sum_{j=1}^{n} w^{\text{D}_{1,i}\text{E}_{j}}\rho_{j}+c_{i}}{r^{\text{M}}},\text{ }$ (12)

$w^{\text{D}_{2,i}\text{M}}\approx\frac{\text{x}^{\text{D}_{2,i}}+\sum_{j=1}^{n} w^{\text{D}_{2,i}\text{E}_{j}}\rho_{j}+c_{i}}{r^{\text{M}}},\text{ }i=1,2,\cdots,n,$ (13)

where

$\begin{matrix} \begin{matrix} r^{\text{M}}= & \left[\text{x}^{\text{M}}+\sum_{i=1}^{n} \left(w^{\text{ME}_{i}}-\frac{w^{\text{VE}_{i}}-w^{\text{VnM}}w^{\text{nME}_{i}}}{1-w^{\text{VnM}}w^{\text{nMV}}}w^{\text{MV}}\rho_{i}\right)-\frac{\text{x}^{\text{V}}-w^{\text{VnM}}\text{x}^{\text{nM}}}{1-w^{\text{VnM}}w^{\text{nMV}}}w^{\text{MV}}\right] \end{matrix} \\ \text{ }\cdot\text{ }\frac{1-w^{\text{VnM}}w^{\text{nMV}}}{1-w^{\text{VnM}}w^{\text{nMV}}-w^{\text{MV}}w^{\text{VM}}} \end{matrix}$ (14)

Equations (9), (12), and (13) together define the complete set of theoretical weights required to achieve E/I balance in PC neurons. Using the resulting weight matrix, we can identify all excitatory and inhibitory pathways targeting both the somatic and dendritic compartments of PCs. These pathway-specific projections provide the quantitative basis for the analysis presented in Figure 9 in Materials and method .

**2. Deduction of synaptic plasticity rules**

We aim to adjust some synaptic weights so that the activity of $N_{\text{PC}}$ PC neurons in the match phase reaches the target firing rate, i.e. $r_{i}^{\text{E}}=\rho_{i}$ $\left(i=1,2,\cdots,N_{\text{PC}}\right)$, which is generally the baseline firing rate. Additionally, we add constraints to the dendritic activity of PC neurons, as they typically do not respond to repeatedly presented matched stimuli due to inhibition from SOM neurons. The training objective for PC dendritic activity is set as , a small positive value close to zero, to reduce the influence of strong onset responses. Therefore, the plasticity rule is based on minimizing the mean square error between the PC neuron activity (Mackwood et al., 2021) and the target value, with the error function defined as follows:

$E(h^{\text{E}},A^{\text{D}_{1}},A^{\text{D}_{2}})=\left\langle\frac{1}{2}\sum_{i=1}^{N_{\text{P} \text{C}}} \left[(h_{i}^{\text{E}}-\rho_{i})^{2}+(A_{i}^{\text{D}_{1}}-\varepsilon)^{2}+(A_{i}^{\text{D}_{2}}-\varepsilon)^{2}\right]\right\rangle_{s}\text{，}$ (15)

where$A_{i}^{\text{D}_{k}}=[I_{\text{D}_{k},i}^{\text{syn}}+c_{i}]_{+}\left(k=1,2\right)$is the dendritic activity of PC neurons. represents the average of all training stimuli.
 To achieve an E/I balance in PC neurons, we can use experience-dependent plasticity training to adjust a subset of inhibitory synapses to minimize the error function $E\left(h^{\text{E}},A^{\text{D}_{1}},A^{\text{D}_{2}}\right)$. This can be accomplished through gradient descent, which optimizes the error function. The specific derivation method is as follows.

All synaptic weights in the equations must remain non-negative to ensure compliance with Dale's law. We achieve this by reparameterizing all synaptic weights in the network using a non-negative function $w=s^{+}\left(v\right)$ and then using the parameter $v$ as the variables to optimize the error function via gradient descent.

We decompose Equation (15) as follows:

(16)

The partial derivative of the error function for the variables

$$v\in\left\{v_{ij}^{\text{EP}},v_{ij}^{\text{ES}},v_{ij}^{\text{D}_{1}\text{S}},v_{ij}^{\text{D}_{2}\text{S}},v_{ij}^{\text{PS}},v_{ij}^{\text{PV}}\right\}$$

is given by:

$\frac{\partial E\left(h^{\text{E}},A^{\text{D}_{1}},A^{\text{D}_{2}}\right)}{\partial v}=\frac{\partial E\left(h^{\text{E}}\right)}{\partial v}+\frac{\partial E\left(A^{\text{D}_{1}}\right)}{\partial v}+\frac{\partial E\left(A^{\text{D}_{2}}\right)}{\partial v}.$ (17)

First, we compute the partial derivative of $E\left(h^{\text{E}}\right)$ with respect to $v$，

$\frac{\partial E\left(h^{\text{E}}\right)}{\partial v}=(h^{\text{E}}-\rho)^{\text{T}}\left\langle\frac{\partial h^{\text{E}}}{\partial v}\right\rangle_{s}.$ (18)

In the above expression, it is necessary first to calculate the partial derivatives of $h^{\text{E}}$ with respect to $v$. When the neurons reach a steady state, their activities satisfy

$\begin{matrix} h^{\text{E}}=\left(1-\lambda_{\text{E}}\right)\left(W^{\text{EE}}r^{\text{E}}-W^{\text{EP}}r^{\text{P}}-W^{\text{ES}}r^{\text{S}}\right) \\ \text{ }+\lambda_{\text{D}}\left(W^{\text{D}_{1}\text{E}}r^{\text{E}}-W^{\text{D}_{1}\text{S}}r^{\text{S}}+W^{\text{D}_{2}\text{E}}r^{\text{E}}-W^{\text{D}_{2}\text{S}}r^{\text{S}}\right)+I^{\text{E}}, \end{matrix}$ (19)

Here, $I^{\text{E}}$ denotes the part of the input to the PC neurons' somas that is independent of $v$. The partial derivative with respect to $v$ is given by

$\begin{matrix} \frac{\partial h^{\text{E}}}{\partial v}=\left[\left(1-\lambda_{\text{E}}\right)W^{\text{EE}}+\lambda_{D}W^{\text{D}_{1}\text{E}}+\lambda_{D}W^{\text{D}_{2}\text{E}}\right]D^{\text{E}}\frac{\partial h^{\text{E}}}{\partial v} \\ \text{ }-\left(1-\lambda_{\text{E}}\right)\left[\frac{\partial W^{\text{EP}}}{\partial v}r^{\text{P}}+W^{\text{EP}}D^{\text{P}}\frac{\partial h^{\text{P}}}{\partial v}+\frac{\partial W^{\text{ES}}}{\partial v}r^{\text{S}}+W^{\text{ES}}D^{\text{S}}\frac{\partial h^{\text{S}}}{\partial v}\right] \\ \text{ }-\lambda_{\text{D}}\left[\frac{\partial W^{\text{D}_{1}\text{S}}}{\partial v}r^{\text{S}}+W^{\text{D}_{1}\text{S}}D^{\text{S}}\frac{\partial h^{\text{S}}}{\partial v}+\frac{\partial W^{\text{D}_{2}\text{S}}}{\partial v}r^{\text{S}}+W^{\text{D}_{2}\text{S}}D^{\text{S}}\frac{\partial h^{\text{S}}}{\partial v}\right], \end{matrix}$ (20)

where $D^{X}$ is a diagonal matrix $D_{ii}^{X}:=\partial r_{i}^{X}/\partial h_{i}^{X}\left(X\in\left\{\text{E},\text{P},\text{S},\text{V}\right\}\right)$.

And $\frac{\partial h^{Y}}{\partial v}\left(Y\in\left\{\text{P},\text{S},\text{V}\right\}\right)$ can be obtained similarly:

$\begin{matrix} \frac{\partial h^{\text{P}}}{\partial v}=\frac{\partial}{\partial v}\left(W^{\text{PE}}r^{\text{E}}-W^{\text{PP}}r^{\text{P}}-W^{\text{PS}}r^{\text{S}}-W^{\text{PV}}r^{\text{V}}+I^{\text{P}}\right) \\ \text{ =}W^{\text{PE}}D^{\text{E}}\frac{\partial h^{\text{E}}}{\partial v}-W^{\text{PP}}D^{\text{P}}\frac{\partial h^{\text{P}}}{\partial v}-\frac{\partial W^{\text{PS}}}{\partial v}r^{\text{S}}-W^{\text{PS}}D^{\text{S}}\frac{\partial h^{\text{S}}}{\partial v}-\frac{\partial W^{\text{PV}}}{\partial v}r^{\text{V}}-W^{\text{PV}}D^{\text{V}}\frac{\partial h^{\text{V}}}{\partial v}, \end{matrix}$ (21)

$\begin{matrix} \frac{\partial h^{\text{S}}}{\partial v}=\frac{\partial}{\partial v}\left(W^{\text{SE}}r^{\text{E}}-W^{\text{SV}}r^{\text{V}}+I^{\text{S}}\right)=W^{\text{SE}}D^{\text{E}}\frac{\partial h^{\text{E}}}{\partial v}-W^{\text{SV}}D^{\text{V}}\frac{\partial h^{\text{V}}}{\partial v}, \end{matrix}$ (22)

$\begin{matrix} \frac{\partial h^{\text{V}}}{\partial v}=\frac{\partial}{\partial v}\left(W^{\text{VE}}r^{\text{E}}-W^{\text{VS}}r^{\text{S}}+I^{\text{V}}\right)=W^{\text{VE}}D^{\text{E}}\frac{\partial h^{\text{E}}}{\partial v}-W^{\text{VS}}D^{\text{S}}\frac{\partial h^{\text{S}}}{\partial v}. \end{matrix}$ (23)

Here, $I^{Y}$ represents the part of the input to $Y$ neurons that is independent of $v$. Substituting Equation (23) into (22) gives:

(24)

where represents the identity matrix. Substituting Equation (24) into Equation (23) yields:

$\begin{matrix} \frac{\partial h^{\text{V}}}{\partial v}=\left(W^{\text{VE}}D^{\text{E}}-W^{\text{VS}}D^{\text{S}}M_{1}\right)\frac{\partial h^{\text{E}}}{\partial v}=:M_{2}\frac{\partial h^{\text{E}}}{\partial v}. \end{matrix}$ (25)

Substituting Equations (24) and (25) into Equation (21) yields:

$\frac{\partial h^{\text{P}}}{\partial v}=\left(W^{\text{PE}}D^{\text{E}}-W^{\text{PS}}D^{\text{S}}M_{1}-W^{\text{PV}}D^{\text{V}}M_{2}\right)\frac{\partial h^{\text{E}}}{\partial v}-W^{\text{PP}}D^{\text{P}}\frac{\partial h^{\text{P}}}{\partial v}-\frac{\partial W^{\text{PS}}}{\partial v}r^{\text{S}}-\frac{\partial W^{\text{PV}}}{\partial v}r^{\text{V}}$ (26)

This can be simplified as follows:

(27)

where,. Equation (20) becomes:

$\begin{matrix} \frac{\partial h^{\text{E}}}{\partial v}=:-M_{5}^{-1}\left[\left(1-\lambda_{\text{E}}\right)\frac{\partial W^{\text{EP}}}{\partial v}r^{\text{P}}+\left(\left(1-\lambda_{\text{E}}\right)\left(\frac{\partial W^{\text{ES}}}{\partial v}-W^{\text{EP}}D^{\text{P}}M_{3}^{-1}\frac{\partial W^{\text{PS}}}{\partial v}\right)\right.\right. \\ \text{ }\left.\left.+\lambda_{\text{D}}\frac{\partial W^{\text{D}_{1}\text{S}}}{\partial v}+\lambda_{\text{D}}\frac{\partial W^{\text{D}_{2}\text{S}}}{\partial v}\right)r^{\text{S}}-\left(1-\lambda_{\text{E}}\right)W^{\text{EP}}D^{\text{P}}R_{3}^{-1}\frac{\partial W^{\text{PV}}}{\partial v}r^{\text{V}}\right], \end{matrix}$ (28)

where

So,

$\begin{matrix} \frac{\partial h^{\text{E}}}{\partial V^{\text{EP}}}=-\left(1-\lambda_{\text{E}}\right)M_{5}^{-1}\frac{\partial W^{\text{EP}}}{\partial V^{\text{EP}}}r^{\text{P}}, \\ \frac{\partial h^{\text{E}}}{\partial V^{\text{ES}}}=-\left(1-\lambda_{\text{E}}\right)M_{5}^{-1}\frac{\partial W^{\text{ES}}}{\partial V^{\text{ES}}}r^{\text{S}}, \\ \frac{\partial h^{\text{E}}}{\partial V^{\text{D}_{1}\text{S}}}=-\lambda_{\text{D}}M_{5}^{-1}\frac{\partial W^{\text{D}_{1}\text{S}}}{\partial V^{\text{D}_{1}\text{S}}}r^{\text{S}}, \\ \frac{\partial h^{\text{E}}}{\partial V^{\text{D}_{2}\text{S}}}=-\lambda_{\text{D}}M_{5}^{-1}\frac{\partial W^{\text{D}_{2}\text{S}}}{\partial V^{\text{D}_{2}\text{S}}}r^{\text{S}}, \\ \frac{\partial h^{\text{E}}}{\partial V^{\text{PS}}}=\left(1-\lambda_{\text{E}}\right)M_{5}^{-1}W^{\text{EP}}D^{\text{P}}M_{3}^{-1}\frac{\partial W^{\text{PS}}}{\partial V^{\text{PS}}}r^{\text{S}}, \\ \frac{\partial h^{\text{E}}}{\partial V^{\text{PV}}}=\left(1-\lambda_{\text{E}}\right)M_{5}^{-1}W^{\text{EP}}D^{\text{P}}M_{3}^{-1}\frac{\partial W^{\text{PV}}}{\partial V^{\text{PV}}}r^{\text{V}}. \end{matrix}$ (29)

where $V^{XY}=(v_{ij}^{XY})_{i,j}$. In the above expression, $M_{5}^{-1}$ and $M_{3}^{-1}$ lead to a highly non-local learning rule, making it difficult to implement in a biologically plausible manner. Given the assumption of sufficiently small synaptic connections, we get

$$\begin{matrix} \left\|\hat{M}_{5}\right\|=\left\|\left(1-\lambda_{\text{E}}\right)\left[W^{\text{EE}}D^{\text{E}}-W^{\text{EP}}D^{\text{P}}M_{4}\right]+\lambda_{\text{D}}(W^{\text{D}_{1}\text{E}}+W^{\text{D}_{2}\text{E}})D^{\text{E}}\right. \\ \text{ }\left.-\left[\left(1-\lambda_{\text{E}}\right)W^{\text{ES}}+\lambda_{\text{D}}(W^{\text{D}_{1}\text{S}}+W^{\text{D}_{2}\text{S}})\right]D^{\text{S}}M_{1}\right\|<1, \end{matrix}$$

and

$\left\|\hat{M}_{3}\right\|=\left\|-W^{\text{PP}}D^{\text{P}}\right\|\text{<1}$.

These yield

$\begin{matrix} M_{5}^{-1}W^{\text{EP}}D^{\text{P}}M_{3}^{-1}=\left(\sum_{k=0}^{\infty} \hat{M}_{5}^{k}\right)W^{\text{EP}}D^{\text{P}}\left(\sum_{k=0}^{\infty} \hat{M}_{3}^{k}\right) \\ \text{ }=W^{\text{EP}}D^{\text{P}}+\hat{M}_{5}W^{\text{EP}}D^{\text{P}}+W^{\text{EP}}D^{\text{P}}\hat{M}_{3}+\left(\sum_{k=1}^{\infty} \hat{M}_{5}^{k}\right)W^{\text{EP}}D^{\text{P}}(\sum_{k=1}^{\infty} \hat{M}_{3}^{k}) \\ \text{ }\approx W^{\text{EP}}D^{\text{P}}, \end{matrix}$ (30)

In the final step, the approximation is achieved by neglecting higher-order correlations among synaptic connections (Mackwood et al., 2021). Furthermore, according to the Neumann series expansion, and , which further simplifies the form of the learning rule.

$\begin{matrix} \frac{\partial E(h^{\text{E}})}{\partial V^{\text{EP}}}=-\left(1-\lambda_{\text{E}}\right)(h^{\text{E}}-\rho)^{\text{T}}\frac{\partial W^{\text{EP}}}{\partial V^{\text{EP}}}r^{\text{P}}, \\ \frac{\partial E(h^{\text{E}})}{\partial V^{\text{ES}}}=-\left(1-\lambda_{\text{E}}\right)(h^{\text{E}}-\rho)^{\text{T}}\frac{\partial W^{\text{ES}}}{\partial V^{\text{ES}}}r^{\text{S}}, \\ \frac{\partial E(h^{\text{E}})}{\partial V^{\text{D}_{1}\text{S}}}=-\lambda_{\text{D}}(h^{\text{E}}-\rho)^{\text{T}}\frac{\partial W^{\text{D}_{1}\text{S}}}{\partial V^{\text{D}_{1}\text{S}}}r^{\text{S}}, \\ \frac{\partial E(h^{\text{E}})}{\partial V^{\text{D}_{2}\text{S}}}=-\lambda_{\text{D}}(h^{\text{E}}-\rho)^{\text{T}}\frac{\partial W^{\text{D}_{2}\text{S}}}{\partial V^{\text{D}_{2}\text{S}}}r^{\text{S}}, \\ \frac{\partial E(h^{\text{E}})}{\partial V^{\text{PS}}}=\left(1-\lambda_{\text{E}}\right)(h^{\text{E}}-\rho)^{\text{T}}W^{\text{EP}}D^{\text{P}}\frac{\partial W^{\text{PS}}}{\partial V^{\text{PS}}}r^{\text{S}}, \\ \frac{\partial E(h^{\text{E}})}{\partial V^{\text{PV}}}=\left(1-\lambda_{\text{E}}\right)(h^{\text{E}}-\rho)^{\text{T}}W^{\text{EP}}D^{\text{P}}\frac{\partial W^{\text{PV}}}{\partial V^{\text{PV}}}r^{\text{V}}, \end{matrix}$ (31)

Similarly, by computing the partial derivative of $A^{\text{D}_{i}}$ with respect to the variable $v$, we obtain

$\frac{\partial A^{\text{D}_{i}}}{\partial v}=\left(W^{\text{D}_{i}\text{E}}D^{\text{E}}-W^{\text{D}_{i}\text{S}}D^{\text{S}}M_{1}\right)\frac{\partial h^{\text{E}}}{\partial v}-\frac{\partial W^{\text{D}_{i}\text{S}}}{\partial v}r^{\text{S}}\approx-\frac{\partial W^{\text{D}_{i}\text{S}}}{\partial v}r^{\text{S}},\text{ }i=1,2.$ (32)

This approximation critically relies on neglecting higher-order nonlinear coupling terms between different synaptic weights, thereby allowing the overall network dynamics to be approximated as a first-order linear system. Consequently, we obtain:

(33)

Summing the corresponding terms in Equations (31) and (33) and neglecting higher-order terms of the synaptic weights, we derive the weight update rules presented in Equations (15)–(17) (see Materials and methods). The weight update rules are $v_{ij}^{XY}=v_{ij}^{XY}+\text{Δ}v_{ij}^{XY}$, and $w_{ij}^{XY}=s^{+}\left(v_{ij}^{XY}\right)$.

**3.Supplementary Figures**

To enhance the completeness of the paper, the following figure has been added.


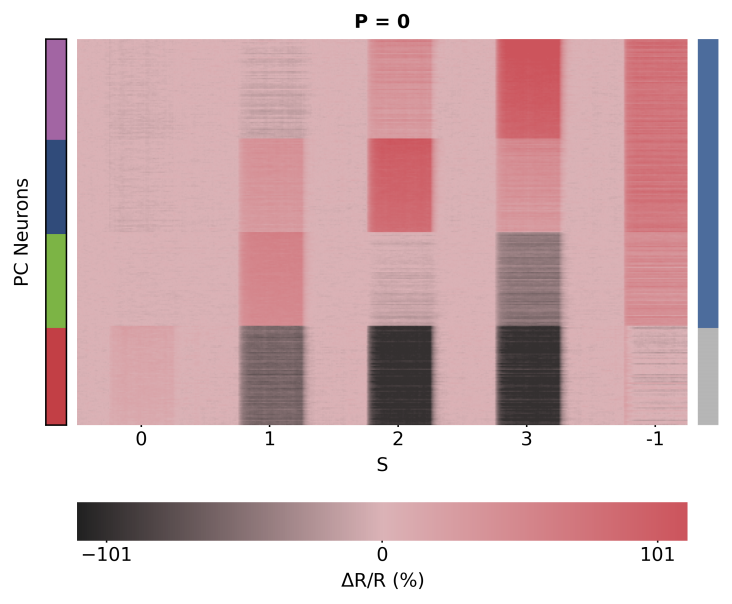


**Supplementary Figure 1.** Model performance without SOM neuron selectivity. This figure illustrates the effect of removing feature selectivity from SOM neurons. In the rightmost column, corresponding to the test phase with prediction $P=0$ and stimulus $S=-1$, although the selectivity coefficient $\gamma$ is nonzero ($\gamma=0.69$), the inhibition exerted by top-down prediction on PC neurons preferring stimulus value 0 remains weak. This highlights the necessity of SOM neuron selectivity for enabling feature-specific top-down suppression.


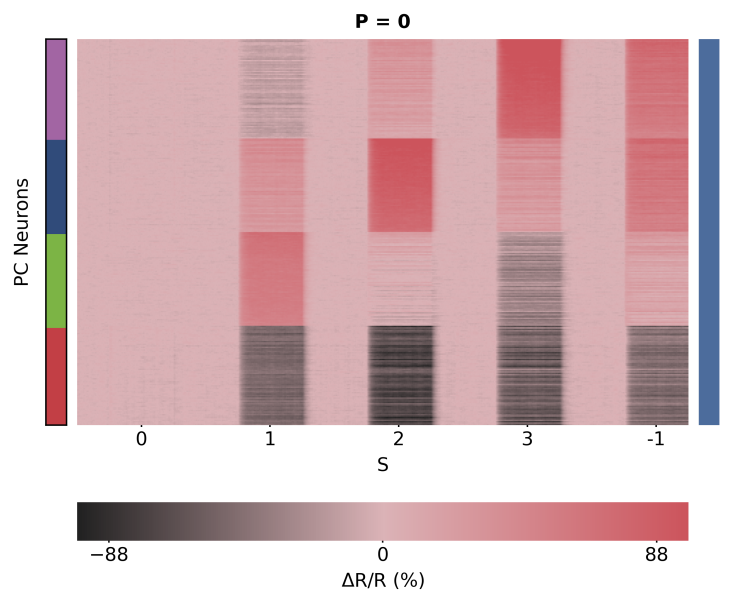


**Supplementary Figure 2.** Heatmap of PC responses under equal tuning widths for SOM and PC neurons. Here, both SOM and PC neurons are assigned the same tuning width ($\sigma_{\text{SOM}}=\sigma_{\text{PC}}=0.8$), unlike the default configuration where SOM neurons have broader tuning. The resulting heatmaps show qualitatively similar prediction error (PE) responses compared to the default model, indicating that the emergence and functional specificity of PE neurons are robust.

To further demonstrate the generality and scalability of the model, we extended our simulations to a two-dimensional feature space. These supplementary visualizations correspond directly to the main one-dimensional results presented in the main text, and confirm that the core computational principles of the model remain valid in higher dimensions.


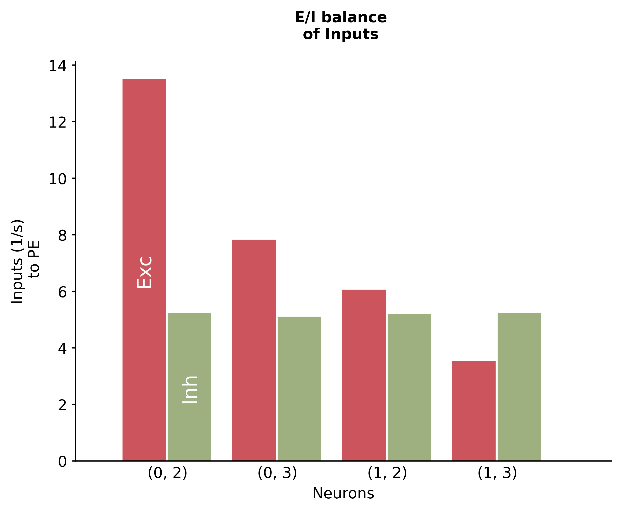

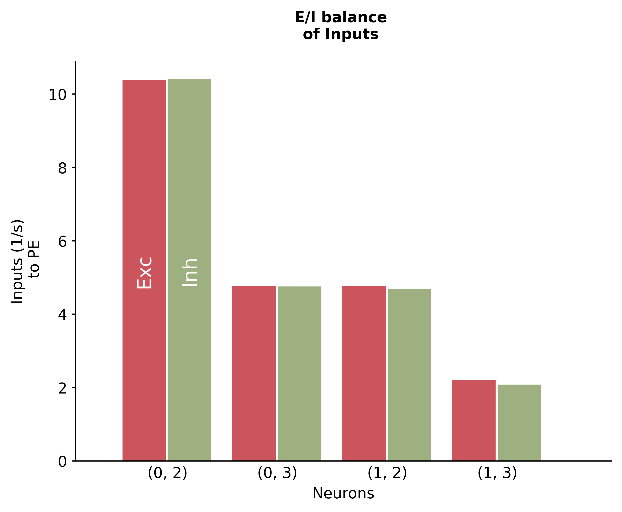


(A) (B)

**Supplementary Figure 3.** Excitation-inhibition (E/I) balance in the two-dimensional prediction error circuit. (A) Before training: E/I inputs to PC neurons are imbalanced across feature combinations. (B) After training: E/I balance is achieved, forming the foundation for PE computation across the two-dimensional space.


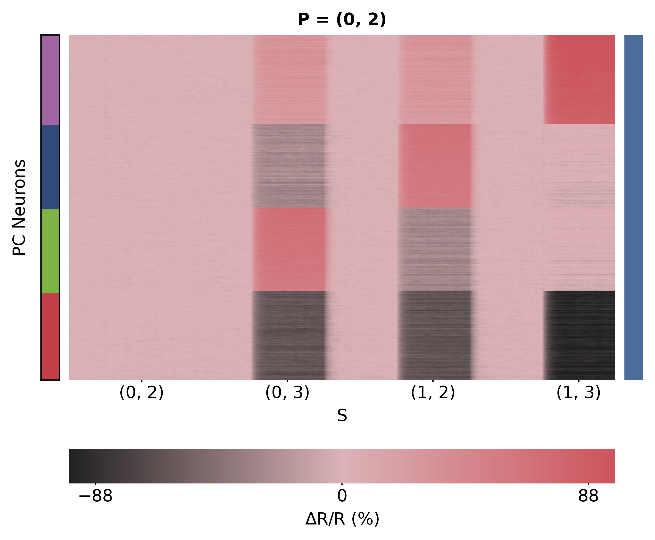


**Supplementary Figure 4.** Activity of two-dimensional prediction error neurons after training. This figure shows the response profile of PE neurons selective for different combinations of the two stimulus features.


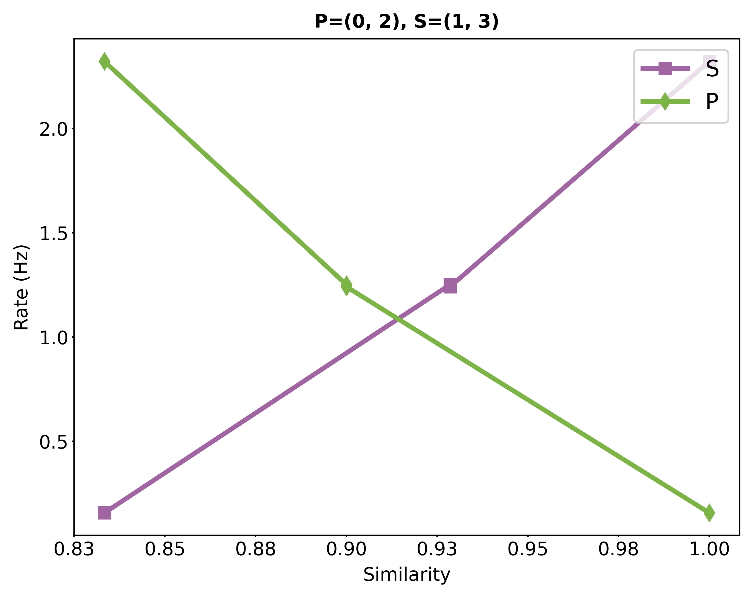


**Supplementary Figure 5.** Neural responses of PE neurons in the two-dimensional model under mismatched conditions. Similar to the one-dimensional case in Figure 3B, PE activity increases with similarity to actual stimuli and decreases with similarity to predicted stimuli, reflecting subtractive mismatch computation across both feature dimensions.
